# Attitudes toward risk and ambiguity in patients with autism spectrum disorder

**DOI:** 10.1186/s13229-017-0162-8

**Published:** 2017-08-16

**Authors:** Junya Fujino, Shisei Tei, Ryu-ichiro Hashimoto, Takashi Itahashi, Haruhisa Ohta, Chieko Kanai, Rieko Okada, Manabu Kubota, Motoaki Nakamura, Nobumasa Kato, Hidehiko Takahashi

**Affiliations:** 1 Medical Institute of Developmental Disabilities Research, Showa University, 6-11-11 Kita-karasuyama, Setagaya-ku, Tokyo, Japan; 2 Department of Psychiatry, Graduate School of Medicine, Kyoto University, 54 Shogoin-Kawaracho, Sakyo-ku, Kyoto, Japan; 3 Institute of Applied Brain Sciences, Waseda University, 2-579-15 Mikajima, Tokorozawa, Saitama, Japan; 4 School of Human and Social Sciences, Tokyo International University, 2509 Matoba, Kawagoe, Saitama, Japan; 5 Department of Language Sciences, Graduate School of Humanities, Tokyo Metropolitan University, 1-1 Minami-Osawa, Hachioji-shi, Tokyo, Japan; 6 Department of Psychiatry, School of Medicine, Showa University, 6-11-11 Kita-karasuyama, Setagaya-ku, Tokyo, Japan; 7 Department of Functional Brain Imaging Research, National Institute of Radiological Sciences, National Institutes for Quantum and Radiological Science and Technology, 4-9-1 Anagawa, Inage-ku, Chiba, Japan; 8 Kanagawa Psychiatric Center, 2-5-1 Serigaya, Yokohama, Kanagawa Japan

**Keywords:** Autism spectrum disorder, Decision-making, Risk, Ambiguity, Uncertainty, Gain, Loss

## Abstract

Although the ability to make optimal decisions under uncertainty is an integral part of everyday life, individuals with autism spectrum disorder (ASD) frequently report that they experience difficulties with this skill. In behavioral economics, researchers distinguish two types of uncertainty to understand decision-making in this setting: risk (known probabilities) and ambiguity (unknown probabilities). However, it remains unclear how individuals with ASD behave under risk and ambiguity, despite growing evidence of their altered decision-making under uncertainty. We therefore extended previous research by studying the attitudes of those with ASD toward risk and ambiguity in both positive and negative contexts (i.e., gain and loss). In gain contexts, no significant difference was observed between the groups in risk attitudes, but ambiguity aversion was attenuated in ASD. In loss contexts, ambiguity attitudes did not significantly differ between the groups, but the ASD participants were less risk-seeking compared with the controls. In addition, insensitivity to the context change under risk and ambiguity in ASD was both significantly associated with poor social skills. These results improve our understanding of altered decision-making under uncertainty by disentangling the attitudes toward risk and ambiguity in ASD individuals. Applying behavioral economic tools may provide insights into the mechanisms underlying behavioral disturbances in ASD.

**Electronic supplementary material:**

The online version of this article (doi:10.1186/s13229-017-0162-8) contains supplementary material, which is available to authorized users.

## Introduction

Decision-making under uncertainty is central to daily functions, because our lives are filled with incomplete, ambiguous, and unpredictable components. Individuals with autism spectrum disorder (ASD), which is characterized by altered social interaction and atypical interests, frequently report having trouble making optimal decisions when faced with such uncertainty [1–3]. This, in turn, can negatively impact their social functioning. Improving our understanding of this problem could provide useful information to help develop practical interventions to improve quality of life in those with ASD.

In recent years, the field of behavioral economics has been expanding rapidly. Researchers in this field distinguish between two types of uncertainty: risk and ambiguity [4, 5]. Under “risk,” the precise probabilities of certain outcomes can be estimated (e.g., gambling on a roulette wheel). However, in real-life situations, the probabilities of outcomes often cannot be estimated (e.g., the likelihood of a terrorist attack is unpredictable), and this latter type of uncertainty is referred to as “ambiguity.” Previous studies of healthy populations have revealed high variability in individual attitudes toward risk and ambiguity, with little correlation between these measures across individuals [5, 6]. This suggests that attitudes toward risk and ambiguity independently contribute to decision-making under uncertainty. Please see Additional file 1 (Supplementary Introduction) for details regarding risk and ambiguity attitudes.

To date, it has been indicated that individual risk and ambiguity attitudes are linked to various types of behavior, including self-insurance and risk-taking behavior [7, 8]. Using tools of behavioral economics, recent studies have begun to investigate risk and ambiguity attitudes with the aim of better understanding altered decision-making under uncertainty in specific clinical populations. For example, a previous study showed that ambiguity aversion was increased in individuals with obsessive-compulsive disorder [9], while another study reported it was attenuated in those with schizophrenia [10]. Findings from these studies laid the groundwork needed to improve the diagnosis and treatment of these psychiatric disorders.

These research findings raise a relevant question: how do individuals with ASD behave under risk and ambiguity? However, to the best of our knowledge, no study has investigated the attitudes of patients with ASD toward uncertainty by clearly distinguishing between risk and ambiguity. Several experimental studies have investigated decision-making under uncertainty in those with ASD, using the Iowa Gambling Task (IGT), which is a well-known behavioral task involving uncertainty [11]. For example, Yechiam et al. reported that patients with ASD did not continue to increase their choices from advantageous decks at the same rate during the IGT when compared with healthy controls [12]. However, an important consideration with the IGT is that the probability distribution is unknown to the participants at the beginning of the test, and they gradually learn this from feedbacks during the task. Therefore, the IGT is a complex measure with elements of decision-making under both risk and ambiguity, where poor performance may partially reflect dysfunctional learning abilities [9, 13].

From a different perspective, De Martino et al. examined the framing effect on monetary decisions in ASD [14]. The framing effect is a well-known decision bias in which people react differently to a particular choice depending on whether it is presented in a positive context (gain) or in a negative one (loss) [15, 16]. Compared with neurotypical participants, adults with ASD demonstrated less susceptibility to the framing effect and made more consistent choices (the results have recently been replicated [2]). The authors proposed that this insensitivity to contextual frame, although enhancing economic rationality, may come at a cost of impaired social skills in ASD [14]. From this perspective, it is crucial to assess attitudes toward risk and ambiguity in both positive and negative contexts (gain and loss) and to investigate the sensitivity to the context change under risk and ambiguity to get a comprehensive picture of these attitudes among patients with ASD.

In this research, we studied risk and ambiguity attitudes in both gain and loss contexts in participants with ASD, using an economic task that clearly distinguished risk and ambiguity containing no feedback learning [6, 7, 9, 17]. Based on the studies mentioned, we hypothesized that ASD participants would exhibit distinctly altered behavioral patterns under risk and ambiguity. In addition, recent studies have shown the association of altered decision-making with the levels of autistic traits [3, 18]. Thus, we also administered the Autism-Spectrum Quotient (AQ) test [19, 20] to quantify ASD-related symptoms and exploratively performed correlation analyses between the task measures and AQ scores among ASD participants.

## Methods

### Participants

Twenty-seven adults with ASD and 27 healthy controls participated in this study. The sample size was determined based on previous studies on psychiatric disorders that used a similar task [9, 10]. Patients were recruited from a database of volunteers who had received a clinical diagnosis of ASD in outpatient units of the Showa University Karasuyama Hospital. The diagnostic procedure to identify patients with ASD was the same as in our previous studies [21–23]. Briefly, at least three experienced psychiatrists and a clinical psychologist assessed all the patients using the criteria of the Diagnostic and Statistical Manual of Mental Disorders, fourth edition text revision (DSM-IV-TR). The assessment consisted of participant interviews about developmental history, present illness, life history, and family history. Patients were also asked to bring suitable informants who had known them in early childhood. This process required approximately 3 h. A diagnosis of ASD was made only when there was a consensus between the psychiatrists and clinical psychologist. At the time of testing, an experienced psychiatrist evaluated psychiatric comorbidity using the Structured Clinical Interview for DSM-IV Axis I Disorders (SCID). No ASD participants satisfied the diagnostic criteria for substance use disorder, bipolar disorder, or schizophrenia. The controls were recruited through advertisements and acquaintances. They did not meet the criteria for any psychiatric disorders according to SCID performed by an experienced psychiatrist. No participants (ASD participants or controls) had any history of head trauma, serious medical or surgical illness, or substance abuse.

The intelligence quotient (IQ) scores of all ASD participants had been evaluated before the study using either the Wechsler Adult Intelligence Scale-Third Edition (WAIS-III) or the WAIS-Revised (WAIS-R). Each participant with ASD was considered to be high functioning because his or her full-scale IQ score was above 80. Although there are several minor changes in WAIS-III from WAIS-R (e.g., more items), the number of core items remained largely unchanged. Therefore, we considered that the WAIS-R and WAIS-III were essentially the same with regard to measuring the full-scale IQ score of individuals with ASD. The IQ scores of the controls were estimated using a Japanese version of the National Adult Reading Test (JART), based on the previous findings that JART successfully predicted the full-scale IQ score in the healthy population [24, 25].

Three ASD participants and one control were excluded from the analyses due to their poor performance in the numeracy test or the decision quality measures, which suggested that they did not understand the task (details are described in Additional file 1: Supplementary Methods). Thus, data from 24 ASD participants and 26 controls were analyzed (age: 20–45 years). In total, 12 ASD participants were administered the following psychotropic drugs: anxiolytics (*n* = 3), antidepressants (*n* = 6), antipsychotics (*n* = 3), antiepileptics (*n* = 2), sleep-inducing drugs (*n* = 6), and other psychotropic drugs (*n* = 3). Although gender, handedness, current smoking status, and estimated full-scale IQ levels were matched between the groups, the control group was slightly younger than the ASD group (Table 1).

**Table 1:** Demographic and clinical characteristics of participants

	Control group	ASD group	Statistics
	(*N* = 26)	(*N* = 24)	*p*
Age (years)	25.7 (6.8)	29.5 (4.3)	0.01^a^
Gender (female)	2 (7.7%)	3 (12.5%)	0.57^b^
Handedness (right)	2 (7.7%)	2 (8.3%)	0.93^b^
Smoking (current smoker)	2 (7.7%)	3 (12.5%)	0.57^b^
Estimated full-scale IQ	105.5 (9.3)	108.0 (12.1)	0.57^a^
AQ total	16.2 (7.0)	34.4 (5.2)	< 0.01^a^
Social skills	2.7 (2.9)	8.8 (3.0)	< 0.01^a^
Details/patterns	3.5 (1.7)	4.7 (2.2)	0.04^a^
Communication/mindreading	1.1 (1.5)	4.3 (1.3)	< 0.01^a^

Standard deviations or percentages in parentheses

*AQ* Autism-Spectrum Quotient, *IQ* intelligence quotient

^a^Mann–Whitney test

^b^Two-tailed chi-square test

This study was approved by the Committee on Medical Ethics of Kyoto University and the institutional review board of Showa University Karasuyama Hospital and was conducted in accordance with The Code of Ethics of the World Medical Association. After a complete description of the study, written informed consent was obtained from all participants.

### Autism-Spectrum Quotient

All participants completed the Japanese version of the Autism-Spectrum Quotient (AQ) test, which has high reliability and validity [19, 20]. The AQ is a brief and self-administered measure that includes items covering both social and nonsocial aspects of behavior and cognition [19, 20]. Therefore, it has been widely used to quantify autistic traits in research and clinical practice. To characterize specific autistic traits of each participant, we computed the three factors: “social skills,” “details/patterns,” and “communication/mindreading” based on the previous studies [26]. Example items are as follows: the social skills factor (“I frequently find that I don’t know how to keep a conversation going” and “I find it hard to make new friends”), the details/patterns factor (“I notice patterns in things all the time” and “I am fascinated by numbers”), and the communication/mindreading factor (“When I’m reading a story, I find it difficult to work out the characters’ intentions” and “I am often the last to understand the point of a joke”). The AQ was scored using the collapsed scoring system [19, 20, 26]. Higher scores for each factor indicated higher autistic traits (e.g., poor social skills).

### Economic task

We modified a task used in the previous studies [6, 7, 9, 17]. The current task consisted of two sessions (gain trials and loss trials sessions). Participants were asked to make a series of choices between pairs of different monetary options. In each trial, participants could choose between a fixed monetary amount and a lottery. The fixed monetary amount did not change from trial to trial, but either the outcome probability or the ambiguity level associated with the lottery option could vary from trial to trial.

**Fig. 1:**
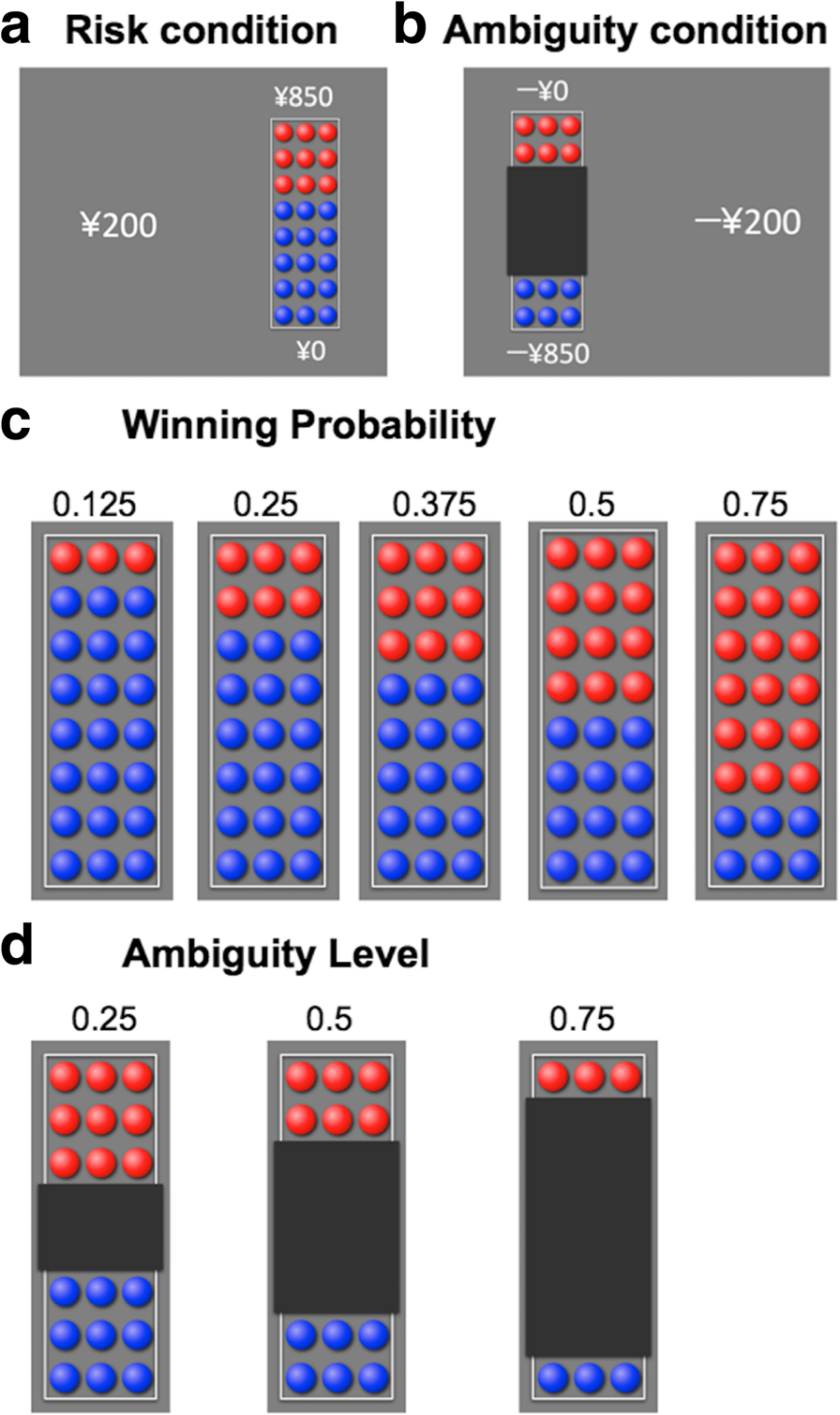
Experimental design. **a** Examples of the stimuli in the risk condition. **b** Examples of the stimuli in the ambiguity condition. In the gain (loss) trials, participants chose between a guaranteed gain (loss) of ¥200 and a lottery that might pay (could yield losses of) more than ¥200 but might also pay ¥0 (could also yield no loss). For instance, as can be seen in **a**, if participants choose the left side, they will win ¥200. If they choose the right side, they will win ¥850 if a red chip is drawn, but they will win ¥0 if a blue chip is drawn. As shown in **b**, if participants choose the left side, they will lose ¥0 if a red chip is drawn, but they will lose ¥850 if a blue chip is drawn. If they choose the right side, they will lose ¥200. **c** The stimuli of the five winning probabilities are shown (red is the winning color). **d** The stimuli of the three ambiguity levels are shown

In the gain trials, participants chose between a guaranteed gain of ¥200 (about $2) and a lottery that might pay more than ¥200 but might also pay ¥0. The lottery appeared on the screen in the form of a bowl containing 24 red- and blue-colored chips. If the bowl was chosen, one chip was drawn, and participants had the right to win the amount allocated to the color of the drawn chip. In risky lotteries, the entire bowl was visible (Fig. 1a). In ambiguous lotteries, we used a black occluder to hide the center of the bowl (Fig. 1b). For instance, as shown in Fig. 1a, if participants choose the left side, they will win ¥200. If they choose the right side, they will win ¥850 if a red chip is drawn, but they will win ¥0 if a blue chip is drawn. In risky lotteries, five winning probabilities were used in the bowl (0.125, 0.25, 0.375, 0.5, and 0.75; Fig. 1c), and in ambiguous lotteries, three ambiguity levels were used in the bowl (0.25, 0.5, and 0.75; Fig. 1d). Probability and ambiguity levels were communicated to the participants using visual displays of lottery bowls before starting the experiment. In addition, we explained that the ambiguous bowls were filled before the experiment began and remained sealed during the experiment, according to the previous studies [6, 7, 9, 17]. It should be noted that the true probability of winning across all ambiguous lotteries was 0.5 because the true probability of winning in each ambiguous lottery was selected at random and fixed throughout a session and because half of all trials paid-off on red and the other half paid-off on blue [6, 7, 9, 17]. Five winning amounts (¥200, ¥350, ¥850, ¥2200, ¥5200) were used per risk and ambiguity level. All lottery situations during the task are shown in Additional file 2 (Tables S1 and S2).

Similarly, the loss trials were performed after the gain trials. In the loss trials, participants chose between a guaranteed loss of ¥200 and a lottery that could yield losses of more than ¥200, but that could also yield no loss (¥0). The probability and ambiguity levels of the lotteries were the same as those in the gain trials. Five losing amounts (− ¥200, − ¥250, − ¥350, − ¥850, and − ¥2200) were used per risk and ambiguity level.

In both gain and loss trial sessions, each unique lottery was presented four times, counterbalancing the winning color and the side it appeared on-screen, resulting in a total of 160 trials, that is, 5 amounts × (5 risk + 3 ambiguity) × 4 = 160 trials (320 trials, summing the gain and loss trials). These trials were separated into four blocks. Each block included 40 trials (i.e., 5 amounts × [5 risk + 3 ambiguity]) and was performed in a pseudorandom order that was unique to each block. During each trial, participants had 6 s to indicate their choice. The interval between successive trials was 2.5 s. Participants could rest between the blocks, and it was up to them to decide when to begin each block. No feedback of the outcome was provided after each choice.

Before starting the task, we performed a numeracy test to assess the participants’ numeracy skills and understanding of numbers (see Additional file 1: Supplementary Methods). All participants were quizzed about how well they understood the task (Additional file 1: Supplementary Methods) and practiced at least once on a short version of the task. Only after they successfully completed the quiz were they allowed to proceed to the experiment. We explained that their winnings were defined referring to the outcomes of three gain trials and three loss trials after they had finished (in reality, we paid a maximum pre-defined participation fee of ¥6000), based on the previous studies [5, 27]. The experiment was presented using E-Prime software (Psychology Software Tools, Inc., Pittsburgh, PA, USA).

### Statistical analyses

Because some of our continuous measures were not normally distributed (Shapiro–Wilk test, *p* < 0.05), we chose Mann–Whitney tests to compare group differences and Spearman’s rank correlations for the correlation analyses. Data were analyzed using SPSS 21 (IBM Corp., Armonk, NY, USA). Statistical significance was set at *p* < 0.05 (two-tailed).

We estimated the participants’ attitudes toward risk and ambiguity based on the previous studies [6, 7, 9, 17]. Under risk, a risk-neutral decision maker would choose the option of the higher expected value (EV; the product of probability and amount) in the gain contexts and the option of the lower EV in the loss contexts. In our task, such a decision maker should choose risky lotteries over the guaranteed payoff 60% of the time in both gain and loss contexts (see Additional file 2: Tables S3 and S4). Participants who chose risky lotteries less or more were termed risk-averse or risk-seeking, respectively. Thus, the risk attitude will be positive for a risk-averse decision maker and negative for a risk-seeking decision maker, when using the following equation:

$$ \mathrm{risk}\  \mathrm{attitude}\ \left(\mathrm{gain},\mathrm{loss}\right)=0.6-\frac{\mathrm{number}\  \mathrm{of}\  \mathrm{risk}\mathrm{y}\  \mathrm{lotteries}\  \mathrm{chosen}}{\mathrm{total}\  \mathrm{number}\  \mathrm{of}\  \mathrm{risk}\mathrm{y}\  \mathrm{lotteries}} $$

Concerning ambiguity, an ambiguity-neutral decision maker would make the same choices regardless of the ambiguity level. Because the range of possible outcome probabilities was centered at 0.5 in all of the ambiguity trials, such a decision-maker should make the same choices in ambiguous trials and in risky trials in which the outcome probability was 0.5. To estimate ambiguity attitudes, we therefore compared each participant’s choices in the ambiguous lotteries to his or her choices in the risky lotteries with a 0.5 outcome probability. Participants who chose ambiguous lotteries less or more often than they chose 0.5 risky lotteries with the same potential reward were termed ambiguity-averse or ambiguity-seeking, respectively. Thus, the ambiguity attitude will be positive for decision makers who are ambiguity-averse and negative for decision-makers who are ambiguity-seeking when using the following equation:

$$ \mathrm{ambiguity}\  \mathrm{attitude}\ \left(\mathrm{gain},\mathrm{loss}\right)=\frac{\mathrm{number}\  \mathrm{of}\ 50\%\mathrm{risky}\  \mathrm{lotteries}\  \mathrm{chosen}}{\mathrm{total}\  \mathrm{number}\  \mathrm{of}\ 50\%\mathrm{risky}\  \mathrm{lotteries}}-\frac{\mathrm{number}\  \mathrm{of}\  \mathrm{ambiguous}\  \mathrm{lotteries}\  \mathrm{chosen}}{\mathrm{total}\  \mathrm{number}\  \mathrm{of}\  \mathrm{ambiguous}\  \mathrm{lotteries}} $$

In addition, we further estimated sensitivity to the context change among participants by taking the difference in their risk and ambiguity attitudes to losses from those to gains. Greater sensitivity was denoted by a larger value.

Finally, we performed correlation analyses between these measures (risk attitudes [gain and loss], ambiguity attitudes [gain and loss], and sensitivity to the context change [risk and ambiguity]) and the three factors of the AQ (social skills, details/patterns, and communication/mindreading) among ASD participants.

## Results

Overall, the participants (*N* = 50 [control 26, ASD 24]) performed the task well, missing an average of only 0.58 ± 1.30 (mean ± standard deviation [SD]) trials. There were no significant differences between the two groups in the decision quality measures, or in reaction time under risk and ambiguity in the gain and loss contexts (see Additional file 1: Supplementary Methods and Additional file 2: Tables S5 and S6). Because the order of sessions in the task was not counterbalanced (the loss trial session followed the gain trial session for all participants), we compared measures related to comprehension/attention and the effect of time on these measures between gain and loss trials in both groups. We found no significant difference between gain and loss trials in terms of decision quality. Details are described in Additional file 1 (Supplementary Results).

### Attitudes toward risk and ambiguity under gain

**Fig. 2:**
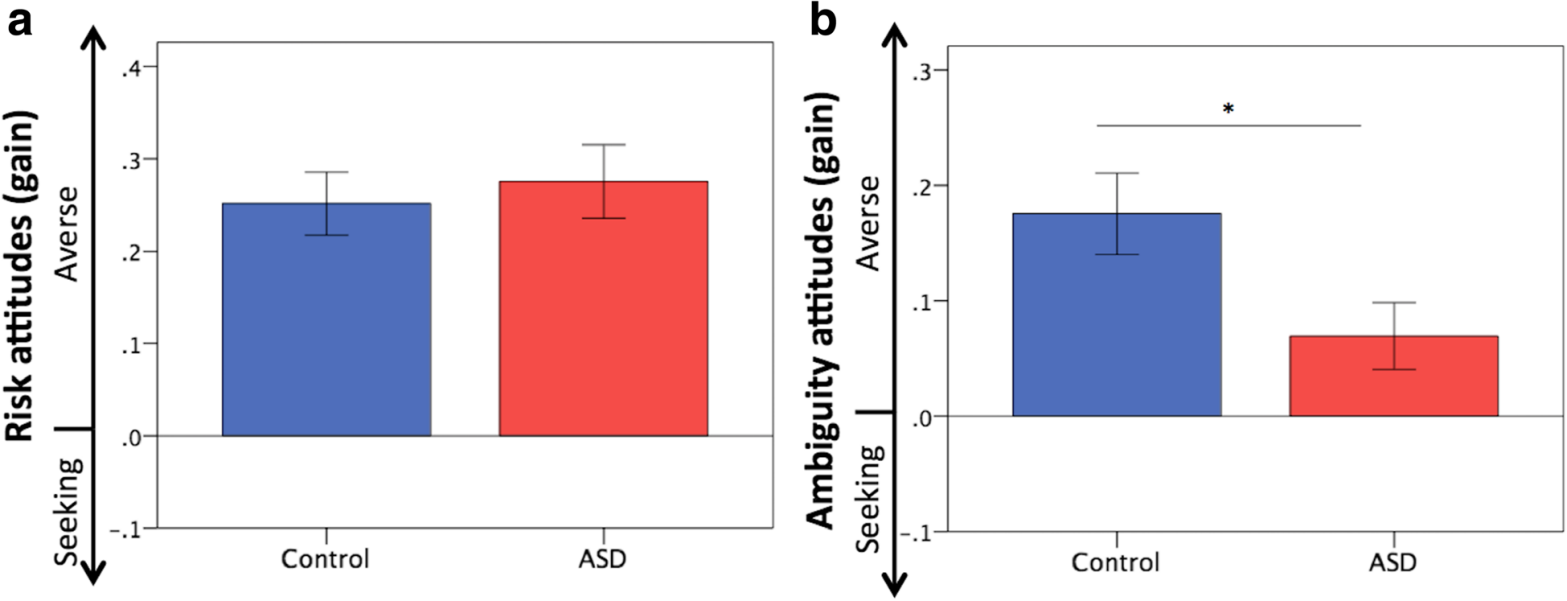
Attitudes toward risk and ambiguity under gain. **a** No significant difference was observed in attitudes toward risk between the groups. **b** The ASD group showed reduced ambiguity aversion compared with the controls. Error bars indicate ± standard errors. *p < 0.05

Figure 2a, b depicts the attitudes toward risk and ambiguity in the gain contexts in both groups. Although no significant difference was found in attitudes toward risk (control 0.25 ± 0.17, ASD 0.28 ± 0.20; *U* = 280.50, *Z* = −0.61, *p* = 0.54, effect size *r* = − 0.09), the ASD group showed attenuated ambiguity aversion (control 0.18 ± 0.18, ASD 0.07 ± 0.14; *U* = 203.50, *Z* = − 2.11, *p* = 0.03, effect size *r* = − 0.30). Because several ASD participants took psychotropic drugs, we compared the behavioral data of ASD participants without psychotropic drugs (*N* = 12) with those of the controls. The analyses did not materially change the results (Additional file 2: Table S7). Please also see Additional file 1 (Supplementary Results), Additional file 3 (Figure S1), and Additional file 4 (Figure S2) for details regarding the distribution of task measures and potential outliers.

### Attitudes toward risk and ambiguity under loss

**Fig. 3:**
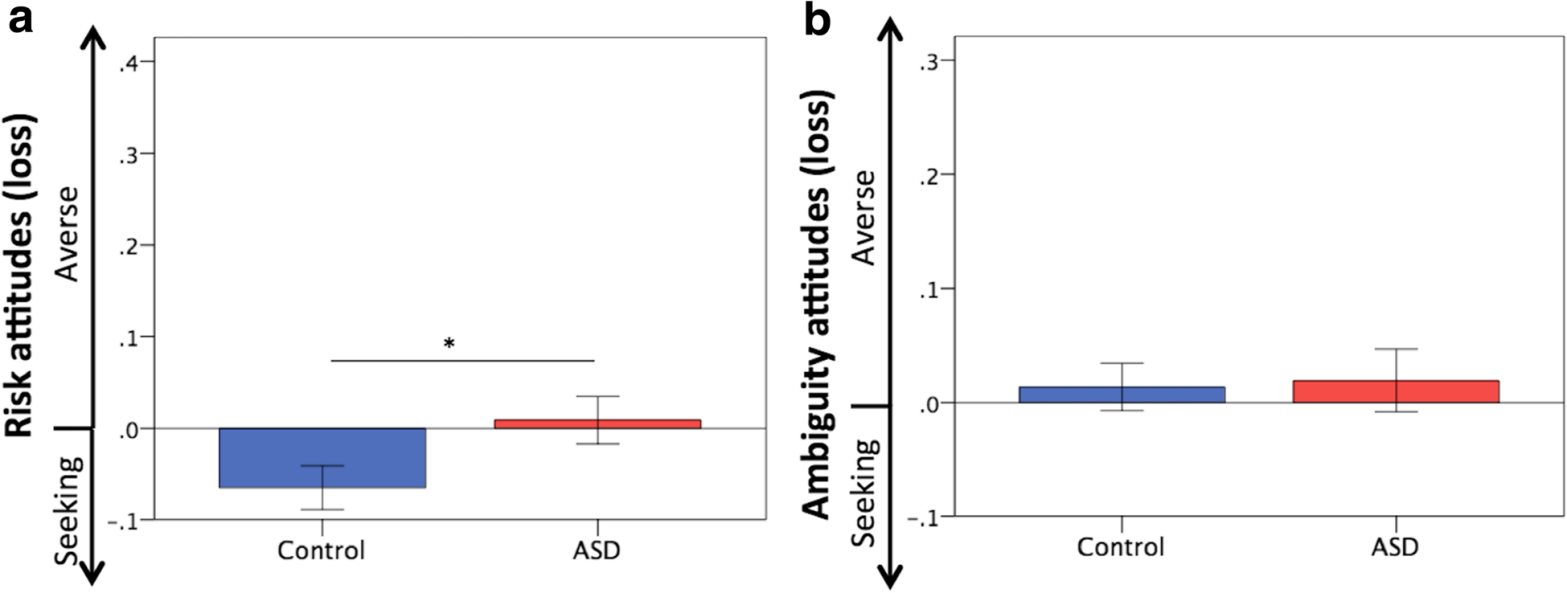
Attitudes toward risk and ambiguity under loss. **a** The ASD participants were less risk-seeking to losses compared with the controls. **b** Ambiguity attitudes to losses did not differ between the groups. Error bars indicate ± standard errors. *p < 0.05

Figure 3a, b depicts the attitudes toward risk and ambiguity in the loss contexts in both groups [please also see Additional file 1 (Supplementary Results), Additional file 3 (Figure S1), and Additional file 4 (Figure S2)]. No significant difference was observed in attitudes toward ambiguity (control 0.01 ± 0.11, ASD 0.02 ± 0.13; *U* = 285.50, *Z* = − 0.52, *p* = 0.61, effect size *r* = − 0.07), but ASD participants were less risk-seeking compared with the controls (control − 0.06 ± 0.12, ASD 0.01 ± 0.13; *U* = 200.50, *Z* = − 2.17, *p* = 0.03, effect size *r* = − 0.31). The analyses after excluding the data on ASD with psychotropic medication did not materially change the results (Additional file 2: Table S7).

### Sensitivity to the context change

We estimated sensitivity to the context change under risk and ambiguity in both groups. Although the difference in sensitivity to the context change under risk did not reach statistical significance between the groups (control 0.32 ± 0.19, ASD 0.27 ± 0.21; *U* = 277.00, *Z* = − 0.68, *p* = 0.50, effect size *r* = − 0.10), this measure under ambiguity was significantly reduced in the ASD group compared with the control group (control 0.16 ± 0.18, ASD 0.05 ± 0.14; *U* = 195.50, *Z* = − 2.27, *p* = 0.02, effect size *r* = − 0.32). The analyses after excluding the data on ASD with psychotropic medication did not materially change the results (Additional file 2: Table S7). Details are described in Additional file 1 (Supplementary Results), Additional file 3 (Figure S1), and Additional file 4 (Figure S2).

### Correlation analyses

**Fig. 4:**
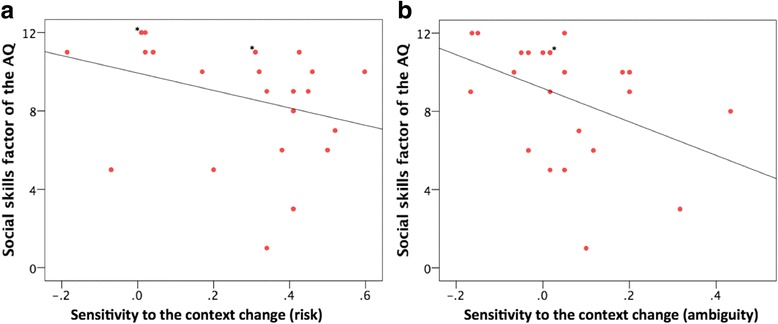
Correlations between sensitivity to the context change under risk and ambiguity and social skills factor of the AQ among ASD participants. **a** Correlations between sensitivity to the context change under risk and the social skills factor of the AQ (*rho* = − 0.44, *p* = 0.03). **b** Correlations between sensitivity to the context change under ambiguity and the social skills factor of the AQ (*rho* = − 0.50, *p* = 0.01). Asterisk, overlapping data point

In the correlation analyses, we found no significant relationship between attitudes toward risk or ambiguity and the AQ factors among ASD participants, except for a negative correlation between risk attitudes to gains and the communication/mindreading factor (*rho* = − 0.43, *p* = 0.03) (Additional file 2: Table S8). Sensitivity to the context change under risk and ambiguity was both significantly and negatively correlated with the social skills factor of AQ (*rho* = − 0.44, *p* = 0.03 and *rho* = − 0.50, *p* = 0.01, respectively; Fig. 4).

## Discussion

To the best of our knowledge, this is the first study to investigate risk and ambiguity attitudes in both gain and loss contexts in participants with ASD. The results provide new insights into altered decision-making under uncertainty that occurs in patients with ASD.

As in previous studies [6, 7, 9, 17], our healthy controls were, on an average, risk averse in the gain context and risk seeking in the loss context; they were also ambiguity averse in the gain context but largely ambiguity neutral in the loss context.

In gain contexts, no significant difference was observed between the groups in risk attitudes, but ambiguity aversion was attenuated in the ASD group. This suggests that they had greater tolerance for options with consequences that had unknown probabilities under positive circumstances. In loss contexts, ambiguity attitudes did not significantly differ between the groups, but the ASD participants were less risk-seeking compared with the controls, indicating that they avoided risk-taking around potential negative outcomes when outcome probabilities were known. These dissociated behavioral patterns, which were altered under risk and ambiguity, might make it difficult to assess behavioral disturbances of this patient group in clinical settings. Previous studies using the IGT have yielded inconsistent results concerning altered decision-making under uncertainty in those with ASD; some studies reported poor performance of IGT in ASD while other studies did not [12, 28–30]. The distinct behavioral patterns altered by risk and ambiguity in our study, along with the mixed results of previous IGT studies, emphasize that it is critical to distinguish risk and ambiguity in order to understand altered decision-making under uncertainty in ASD clearly. We found no significant relationship between either the risk or the ambiguity attitudes and the AQ factors, except for the negative correlation between risk attitudes to gains and the communication/mindreading factor. Therefore, we could not infer the mechanisms underlying the altered attitudes toward risk and ambiguity in ASD. However, our findings should provide an impetus for future neuroimaging studies that elucidate the mechanisms of altered attitudes toward risk and ambiguity in those with ASD.

It is important to note that sensitivity to the context change under both risk and ambiguity was significantly and negatively correlated with the social skills factor of the AQ in ASD participants (although the difference in this measure under risk did not reach statistical significance between the groups, it served as important individual difference variables within the ASD group). It is well known that individuals react to a particular choice in a different manner under risk depending on whether it is presented as a gain or as a loss (referred to as the “framing effect”) [15, 16]. Furthermore, a recent study demonstrated that the framing effect also existed during decision-making under ambiguity [31]. This effect is economically irrational but seems to play a key role in making optimal decisions under uncertain situations [15]. Previous studies have examined the framing effect on monetary decisions in individuals with ASD [2, 14]. These studies have shown that ASD individuals are less susceptible to the framing effect and make more consistent choices, and the authors have proposed that ASD confers enhanced logical consistency, which clinically presents as reduced behavioral flexibility. More recently, Farmer et al. compared a series of choices regarding consumer products between ASD participants and controls [18] and showed that participants’ preferences between a given pair of options frequently switched when the third item in the set was changed. This tendency was reduced among ASD participants, indicating more consistent and conventionally rational choices [a comparison of people with low- vs high-levels of autistic traits assessed by AQ (drawn from the general population) also revealed a weaker version of the same effect]. The authors have proposed that the price ASD individuals pay for this resistance to contextual influence may be a reduction in the potentially adaptive updating of beliefs about optimum choices that come from using local comparisons to inform decision-making. Taken together, our results, using a different experimental paradigm, supported these previous findings [2, 14, 18] and suggested that a difficulty to adapt behavioral strategies according to the valence of the context under both risk and ambiguity might be related with poor social skills in individuals with ASD.

As for the clinical implications of this study, our findings support the notion that behavioral economics is a promising method for objectively evaluating behavioral disturbance in patients with psychiatric disorders [9, 17]. Rodgers and colleagues have elegantly demonstrated that intolerance of uncertainty plays an important role in understanding anxiety symptoms in ASD [32–36]. Although the jargon used in psychology and economics is not necessarily identical and careful interpretation is needed, a previous study reported a possible link between ambiguity intolerance and ambiguity aversion in healthy population [37]. The behavioral economics tools are relatively affordable and easy to administer, and thus, applying these tools may provide insights into the mechanisms underlying behavioral disturbances under uncertain environment in ASD.

There are several limitations to this study. First, half the individuals with ASD were administered psychotropic medication, which means that we cannot exclude the possibility of a medication effect. For example, previous studies have shown that dopaminergic and serotoninergic agents influence risk-taking behaviors [38]. In addition, Beninger et al. found an association between antipsychotic drug use status and better performance on the gambling task in patients with schizophrenia [39]. Unfortunately, the medicated participants with ASD in our study were administered different types of psychotropic drugs, hampering further analysis of the effect of medication on our behavioral results. However, we still found significant differences between the groups with regard to the three measures (i.e., risk attitudes [loss], ambiguity attitudes [gain], and sensitivity to the context change [ambiguity]) when analyzing the data, excluding those of the ASD participants administered psychotropic medication. Our findings should be replicated in future research that recruits more individuals with ASD who are not taking medication. Second, the order of sessions in our task was not counterbalanced (the loss trial session followed the gain trial session for all participants). Thus, factors related to the order of testing (e.g., fatigue) have the possibility of causing confounding. However, to minimize the influence of fatigue on the task performance, we allowed sufficient rest time between each block. The experimenter also observed participants during the task, and the post-task interview confirmed that their concentration throughout the task was good. Furthermore, we found no significant difference between gain and loss sessions in terms of decision quality for both groups. Before data collection, we took steps to ensure that task instructions were understood; in these steps, the participants were required to pass the numeracy test and comprehension quizzes. Thus, we believe that our study can provide a good foundation for future studies. Third, although the difference was small, the healthy controls were younger. A previous study investigated risk and ambiguity attitudes in both gain and loss contexts in healthy subjects aged 12–90 years and found no significant differences in these attitudes between the young adults group (21–25 years) and the midlife adults group (30–50 years) [6]. Therefore, given the age range of our sample (20–45 years), we believe that our findings will be useful for obtaining a better understanding of decision-making under uncertainty in ASD. Fourth, emotional factors, including interoception, have recently been reported to play a key role in decision-making [2, 40, 41]. For example, Shah et al. have demonstrated that framing effects are associated with interoception and alexithymia in the neurotypical population but that emotional and interoceptive signals have less impact on decision-making processes in ASD [2]. However, we did not measure factors, such as alexithymia, in the current study. Future studies should comprehensively take social and emotional factors into account and investigate the contribution of such factors in risk and ambiguity attitudes in ASD. Fifth, because of the exploratory nature of this study, no correction was applied for multiple comparisons during the group comparisons of the task measures (Mann–Whitney tests) and the correlation analyses between these measures and the AQ factors. Thus, our preliminary findings should be interpreted cautiously and need to be carefully replicated in future research. Nevertheless, the current results may serve as an indication of the fruitfulness of research in this area and motivate future research on a wider scale.

## Conclusions

In conclusion, the current results extend previous findings by disentangling the attitudes toward risk and ambiguity in individuals with ASD. Applying behavioral economic tools may provide insights into the mechanisms underlying behavioral disturbances in ASD.

## Additional files

Additional file 1: Supporting information concerning the task measures. Detailed explanations regarding risk and ambiguity attitudes are included in the Supplementary Introduction. Details concerning the exclusion criteria, numeracy test, quiz, and decision quality measures are described in the Supplementary Methods. The distribution of the task measures, potential outliers, and decision quality in gain and loss trials are explained in the Supplementary Results. (DOCX 1737 kb)

Additional file 2: Tables showing the details of the task measures. **Table S1.** All lottery situations in the gain trials. Table S2. All lottery situations in the loss trials. Table S3. Probability of choosing a lottery for a risk-neutral decision maker in the gain contexts. Table S4. Probability of choosing a lottery for a risk-neutral decision maker in the loss contexts. Table S5. Measures of decision quality. Table S6. Reaction time (ms) in risk and ambiguity conditions. **Table S7.** Results of additional analyses between ASD participants without psychotropic medication and controls. Table S8. Correlation coefficients between risk and ambiguity attitudes and the three factors of the Autism-Spectrum Quotient (AQ) among ASD participants. (DOCX 84 kb)

Additional file 3: Distribution of the task measures (**Figure S1.**) **Figure S1.** depicts the distribution of each of the task measures (risk attitudes [gain and loss], ambiguity attitudes [gain and loss], and sensitivity to the context change [risk and ambiguity]). Because some of the task measures were not normally distributed (Shapiro–Wilk test, *p* < 0.05), we chose Mann–Whitney tests to compare group differences. Concerning the task measures that were normally distributed, we also compared the group difference using two-sample t-tests, which did not materially change the results. (TIFF 1149 kb)

Additional file 4 Analyses of the task measures after excluding the potential outliers (**Figure S2.**) To confirm our conclusion of this study, we also reanalyzed the group comparison of the task measures (risk attitudes [gain and loss], ambiguity attitudes [gain and loss], and sensitivity to the context change [risk and ambiguity]) after excluding the potential outliers of each task measure (> 2 SD from the group mean). These analyses did not materially change the results. (TIFF 1368 kb)

## Abbreviations

AQ: Autism-Spectrum Quotient
ASD: Autism spectrum disorder
DSM-IV-TR: Diagnostic and Statistical Manual of Mental Disorders fourth edition text revision
EV: Expected value
IGT: Iowa Gambling Task
IQ: Intelligence quotient
JART: Japanese version of the National Adult Reading Test
SCID: Structured Clinical Interview for DSM-IV Axis I Disorders
SD: Standard deviation
WAIS-III: Wechsler Adult Intelligence Scale-Third Edition
WAIS-R: Wechsler Adult Intelligence Scale-Revised

## References

[1] Luke L, Clare IC, Ring H, Redley M, Watson P (2012). Decision-making difficulties experienced by adults with autism spectrum conditions. Autism.

[2] Shah P, Catmur C, Bird G (2016). Emotional decision-making in autism spectrum disorder: the roles of interoception and alexithymia. Mol Autism..

[3] Robic S, Sonie S, Fonlupt P, Henaff M-A, Touil N, Coricelli G (2015). Decision-making in a changing world: a study in autism spectrum disorders. J Autism Dev Disord.

[4] Camerer C, Weber M (1992). Recent developments in modeling preferences: uncertainty and ambiguity. J Risk Uncertainty.

[5] Levy I, Snell J, Nelson AJ, Rustichini A, Glimcher PW (2010). Neural representation of subjective value under risk and ambiguity. J Neurophysiol.

[6] Tymula A, Belmaker LAR, Ruderman L, Glimcher PW, Levy I (2013). Like cognitive function, decision making across the life span shows profound age-related changes. Proc Natl Acad Sci U S A.

[7] Tymula A, Belmaker LAR, Roy AK, Ruderman L, Manson K, Glimcher PW (2012). Adolescents’ risk-taking behavior is driven by tolerance to ambiguity. Proc Natl Acad Sci U S A.

[8] Alary D, Gollier C, Treich N (2013). The effect of ambiguity aversion on insurance and self-protection. Econ J.

[9] Pushkarskaya H, Tolin D, Ruderman L, Kirshenbaum A, Kelly JM, Pittenger C (2015). Decision-making under uncertainty in obsessive–compulsive disorder. J Psychiatr Res.

[10] Fujino J, Hirose K, Tei S, Kawada R, Tsurumi K, Matsukawa N (2016). Ambiguity aversion in schizophrenia: an fMRI study of decision-making under risk and ambiguity. Schizophr Res.

[11] Bechara A, Damasio AR, Damasio H, Anderson SW (1994). Insensitivity to future consequences following damage to human prefrontal cortex. Cognition.

[12] Yechiam E, Arshavsky O, Shamay-Tsoory SG, Yaniv S, Aharon J (2010). Adapted to explore: reinforcement learning in autistic spectrum conditions. Brain Cogn.

[13] Buckert M, Schwieren C, Kudielka BM, Fiebach CJ (2014). Acute stress affects risk taking but not ambiguity aversion. Front Neurosci.

[14] De Martino B, Harrison NA, Knafo S, Bird G, Dolan RJ (2008). Explaining enhanced logical consistency during decision making in autism. J Neurosci.

[15] De Martino B, Kumaran D, Seymour B, Dolan RJ (2006). Frames, biases, and rational decision-making in the human brain. Science.

[16] Tversky A, Kahneman D (1981). The framing of decisions and the psychology of choice. Science.

[17] Ruderman L, Ehrlich DB, Roy A, Pietrzak RH, Harpaz-Rotem I, Levy I (2016). Posttraumatic stress symptoms and aversion to ambiguous losses in combat veterans. Depress Anxiety.

[18] Farmer G, Baron-Cohen S, Skylark W (2017). People with autism spectrum conditions make more consistent decisions. Psychol Sci..

[19] Baron-Cohen S, Wheelwright S, Skinner R, Martin J, Clubley E (2001). The autism-spectrum quotient (AQ): evidence from asperger syndrome/high-functioning autism, malesand females, scientists and mathematicians. J Autism Dev Disord.

[20] Wakabayashi A, Baron-Cohen S, Wheelwright S, Tojo Y (2006). The Autism-Spectrum Quotient (AQ) in Japan: a cross-cultural comparison. J Autism Dev Disord.

[21] Itahashi T, Yamada T, Watanabe H, Nakamura M, Ohta H, Kanai C (2015). Alterations of local spontaneous brain activity and connectivity in adults with high-functioning autism spectrum disorder. Mol Autism..

[22] Itahashi T, Yamada T, Nakamura M, Watanabe H, Yamagata B, Jimbo D (2015). Linked alterations in gray and white matter morphology in adults with high-functioning autism spectrum disorder: a multimodal brain imaging study. Neuroimage Clin.

[23] Yamada T, Itahashi T, Nakamura M, Watanabe H, Kuroda M, Ohta H (2016). Altered functional organization within the insular cortex in adult males with high-functioning autism spectrum disorder: evidence from connectivity-based parcellation. Mol Autism..

[24] Matsuoka K, Kim Y (2006). Japanese Adult Reading Test (JART).

[25] Matsuoka K, Uno M, Kasai K, Koyama K, Kim Y (2006). Estimation of premorbid IQ in individuals with Alzheimer’s disease using Japanese ideographic script (Kanji) compound words: Japanese version of national adult reading test. Psychiatry Clin Neurosci.

[26] Hurst R, Mitchell J, Kimbrel N, Kwapil T, Nelson-Gray R (2007). Examination of the reliability and factor structure of the Autism Spectrum Quotient (AQ) in a non-clinical sample. Pers Individ Dif.

[27] Tanaka Y, Fujino J, Ideno T, Okubo S, Takemura K, Miyata J (2014). Are ambiguity aversion and ambiguity intolerance identical? A neuroeconomics investigation. Front Psychol.

[28] South M, Chamberlain PD, Wigham S, Newton T, Le Couteur A, McConachie H (2014). Enhanced decision making and risk avoidance in high-functioning autism spectrum disorder. Neuropsychology.

[29] Johnson SA, Yechiam E, Murphy RR, Queller S, Stout JC (2006). Motivational processes and autonomic responsivity in Asperger’s disorder: evidence from the Iowa Gambling Task. J Int Neuropsychol Soc.

[30] Mussey JL, Travers BG, Klinger LG, Klinger MR (2015). Decision-making skills in ASD: performance on the Iowa Gambling Task. Autism Res.

[31] Osmont A, Cassotti M, Agogué M, Houdé O, Moutier S (2015). Does ambiguity aversion influence the framing effect during decision making?. Psychon Bull Rev.

[32] Boulter C, Freeston M, South M, Rodgers J (2014). Intolerance of uncertainty as a framework for understanding anxiety in children and adolescents with autism spectrum disorders. J Autism Dev Disord.

[33] Chamberlain PD, Rodgers J, Crowley MJ, White SE, Freeston MH, South M (2013). A potentiated startle study of uncertainty and contextual anxiety in adolescents diagnosed with autism spectrum disorder. Mol Autism.

[34] Rodgers J, Glod M, Connolly B, McConachie H (2012). The relationship between anxiety and repetitive behaviours in autism spectrum disorder. J Autism Dev Disord.

[35] Wigham S, Rodgers J, South M, McConachie H, Freeston M (2015). The interplay between sensory processing abnormalities, intolerance of uncertainty, anxiety and restricted and repetitive behaviours in autism spectrum disorder. J Autism Dev Disord.

[36] Keefer A, Kreiser NL, Singh V, Blakeley-Smith A, Duncan A, Johnson C, Klinger L, Meyer A, Reaven J, Vasa RA. Intolerance of uncertainty predicts anxiety outcomes following CBT in youth with ASD. J Autism Dev Disord. 2016; in press 10.1007/s10803-016-2852-z 27405445

[37] Sherman R (1974). The psychological difference between ambiguity and risk. Q J Econ.

[38] Rogers RD (2011). The roles of dopamine and serotonin in decision making: evidence from pharmacological experiments in humans. Neuropsychopharmacology.

[39] Beninger RJ, Wasserman J, Zanibbi K, Charbonneau D, Mangels J, Beninger BV (2003). Typical and atypical antipsychotic medications differentially affect two nondeclarative memory tasks in schizophrenic patients: a double dissociation. Schizophr Res.

[40] Shah P, Catmur C, Bird G (2017). From heart to mind: linking interoception, emotion, and theory of mind. Cortex.

[41] Ondobaka S, Kilner J, Friston K (2017). The role of interoceptive inference in theory of mind. Brain Cogn.

