# Supplementary material for: Attitudes toward risk and ambiguity in patients with autism spectrum disorder
Source: Mol Autism. 2017 Aug 16;8:45. doi: 10.1186/s13229-017-0162-8 (PMC5559781; doi:10.1186/s13229-017-0162-8)
Supplement: Supplementary file 1 — Supporting information concerning the task measures. Detailed explanations regarding risk and ambiguity attitudes are included in the Supplementary Introduction. Details concerning the exclusion criteria, numeracy test, quiz, and decision quality measures are described in the Supplementary Methods. The distribution of the task measures, potential outliers, and decision quality in gain and loss trials are explained in the Supplementary Results. (DOCX 1737 kb) [file 13229_2017_162_MOESM1_ESM.docx]

**Additional file 1**

**Supplementary Introduction**

*Risk and ambiguity attitudes*

Previous studies of healthy populations have revealed high variability in individual attitudes toward risk and ambiguity [1, 2]. Risk aversion has been defined as the tendency to prefer high probabilities of low winning amounts to low probabilities of high winning amounts, even when the expected value (EV, the product of probability and amount) is lower for the former option [1–3]. For example, risk-averse people will take a certain $100 win over a 50% chance of winning $240, although the EV of the former option is lower than that of the latter ($100 vs $120). By contrast, risk-seeking individuals prefer low probabilities of high-winning amounts to high probabilities of low-winning amounts, even when the EV is lower for the former option [1, 2]. Risk-neutral preferences are neither risk averse nor risk seeking.

Ambiguity aversion has been defined as the tendency to prefer risk over ambiguity [1, 4], and can be illustrated using the Ellsberg paradox [5]. Suppose there are two bowls that are each filled with a mixture of 24 chips colored red and blue. One bowl has exactly 12 red chips and 12 blue chips (the risky bowl), but the composition of the other bowl is unknown to the participants (the ambiguous bowl). Participants are asked to select one bowl and are told that if a red chip is drawn, they qualify for a predefined payoff. For the risky bowl, the probability of drawing a red chip is 0.5. For the ambiguous bowl, the probability of drawing a red chip is unknown, but the probability of winning is also 0.5. Instead of 24 chips, imagine there are only 2. In the risky bowl, there is 1 red and 1 blue. In the ambiguous bowl, one-third of the time participants would be picking out of the bowl with 2 red chips. Another third of the time, 2 blue chips. And another third of the time, the bowl has 1 of each. Nevertheless, previous studies have shown that most individuals choose the risky bowl, even if its payoff is lower than that of the ambiguous one, in gain contexts [1, 4, 5]. In contrast to this, ambiguity-seeking individuals are believed to prefer ambiguity over risk [1, 2]. Ambiguity neutral preferences are neither ambiguity averse nor ambiguity seeking.

**Supplementary Methods**

*Exclusion criteria*

We excluded participants from the analyses if their score in the numeracy test or decision quality measure 2 was more than 2 SD from the mean, indicating that they did not understand the task. The exclusion criteria of the decision quality measure 1 were over 0.5 based on previous studies [6]. Details are described in the Numeracy test and Decision quality measures section.

We performed the quiz via a face-to-face interview with the experimenter to promote the participants’ understanding of the task. Thus, the nature of our quiz was different from the other two measures (numeracy and decision quality). Two ASD participants and two controls misunderstood one or two points, but their misunderstanding was immediately corrected after being shown the correct answers. Therefore, we did not exclude these participants from the analyses.

*Numeracy test*

1. If the chance of winning a lottery is 20 in 100, what percent of people will win the lottery?

_________________ (correct answer [20%])

1. What is 25% of 1000?

_________________ (correct answer [250])

1. Which of the following percentages is the highest?
2. 5%
3. 20%
4. 15%

________________ (correct answer [b])

1. Which of the following disease represents the biggest risk of getting?
2. The chance of getting the disease is 3 in 100
3. The chance of getting the disease is 3 in 10
4. The chance of getting the disease is 3 in 1000

________________ (correct answer [b])

1. If \6000 is divided among 3 people, how much will each of them get?

________________ (correct answer [\2000])

The score on the numeracy test was the total number of correct answers. The scores of all participants (*N* = 54) were 4.83 ± 0.42 (mean ± standard deviation [SD]) (control 4.78 ± 0.42, autism spectrum disorder [ASD] 4.89 ± 0.42, *p* = 0.15). The score of one ASD participant was extremely low compared with the overall average (more than 3 SD below the mean), which suggested that he did not have the basic numeracy skills necessary to understand the task (there were no other participants who scored more than 2 SD away from the mean). Thus, we excluded him from the analysis.

*Quiz for gain trials*

1. How much will you win if you choose the left side in this trial?


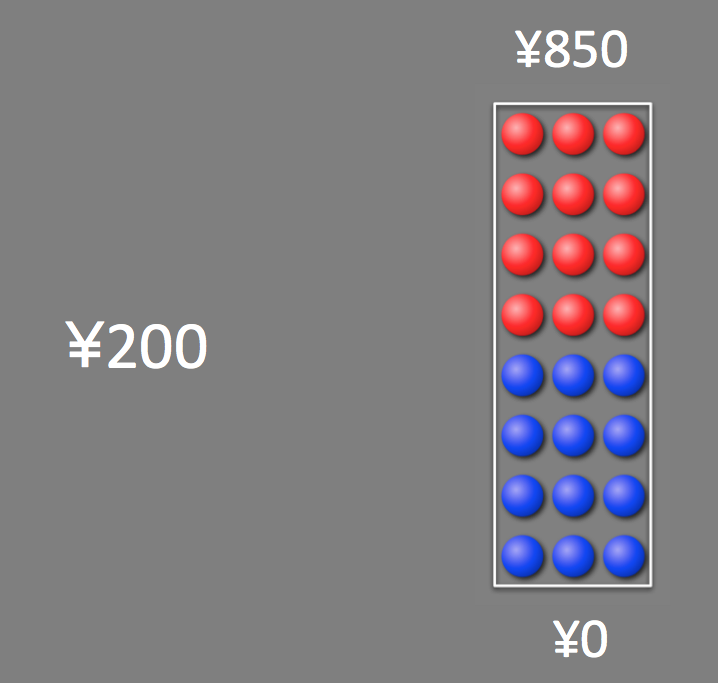


(correct answer [\200])


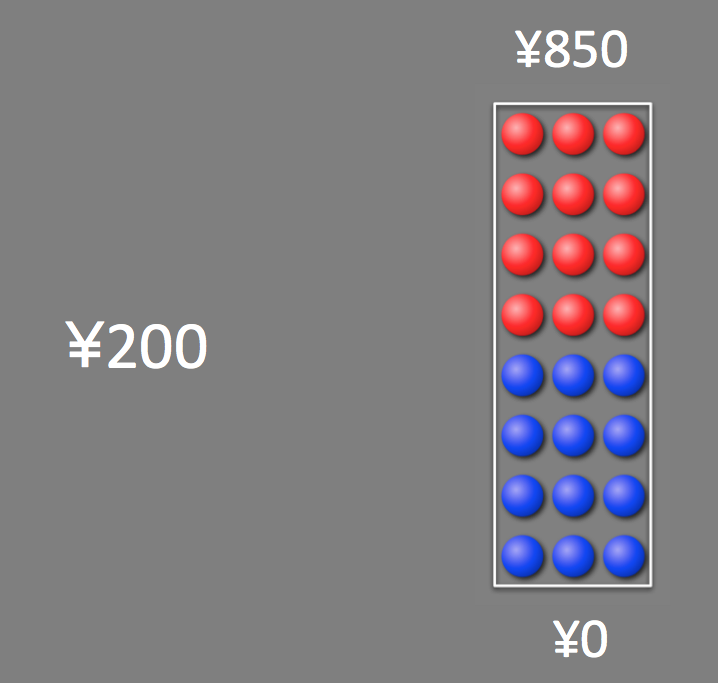
2. How much will you win if you choose the right side and a red chip is drawn in this trial?

(correct answer [\850])


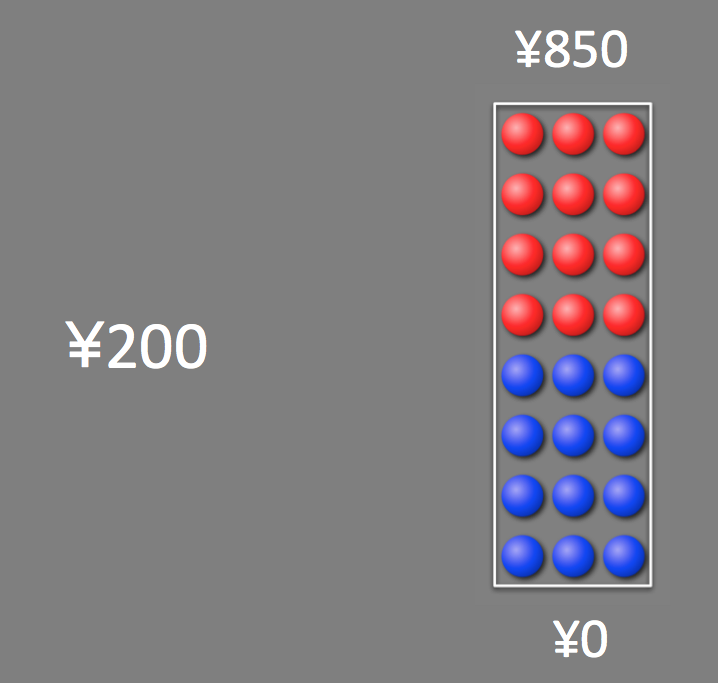
3. How much will you win if you choose the right side and a blue chip is drawn in this trial?

(correct answer [\0])


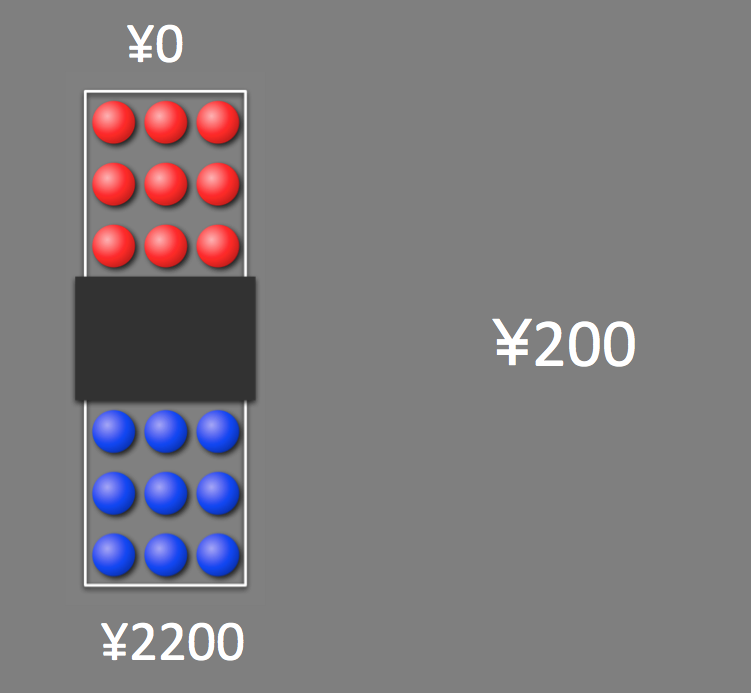
4. How much will you win if you choose the left side and a blue chip is drawn in this trial?

(correct answer [\2200])

5. How many red chips are there in this bowl?

1.
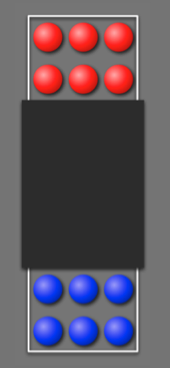
0
2. 24
3. 6
4. between 6 and 18
5. 18

(correct answer [d])


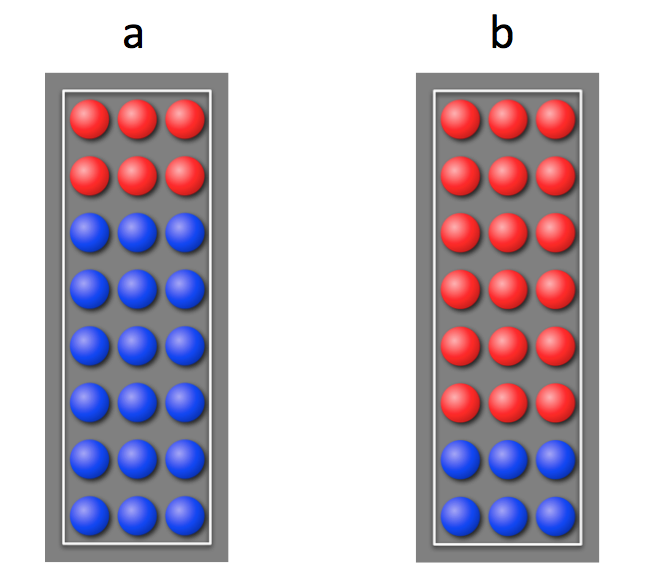
6. From which bowl is a red chip more likely to be drawn?

(correct answer [b])

7. Which option is more likely to get you a win in this trial?


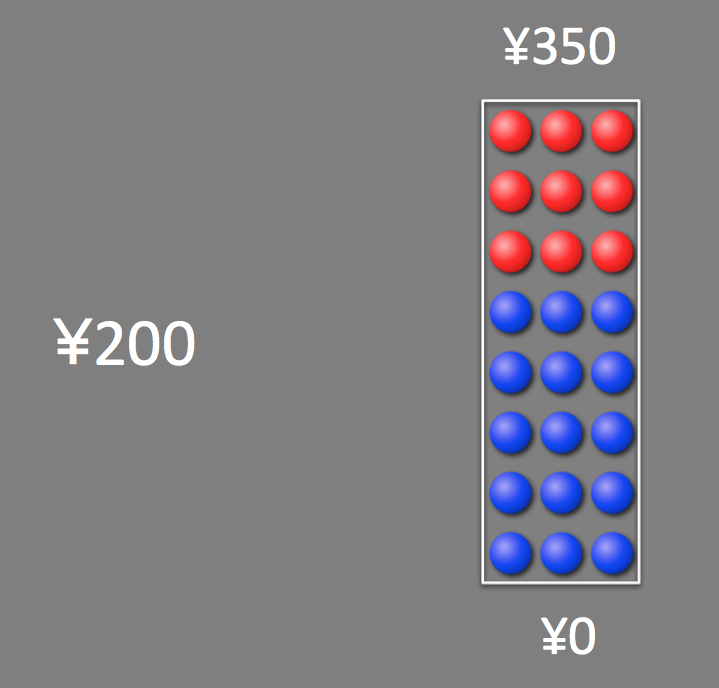


(correct answer [left])

8. Which option is more likely to get you \200 in this trial?


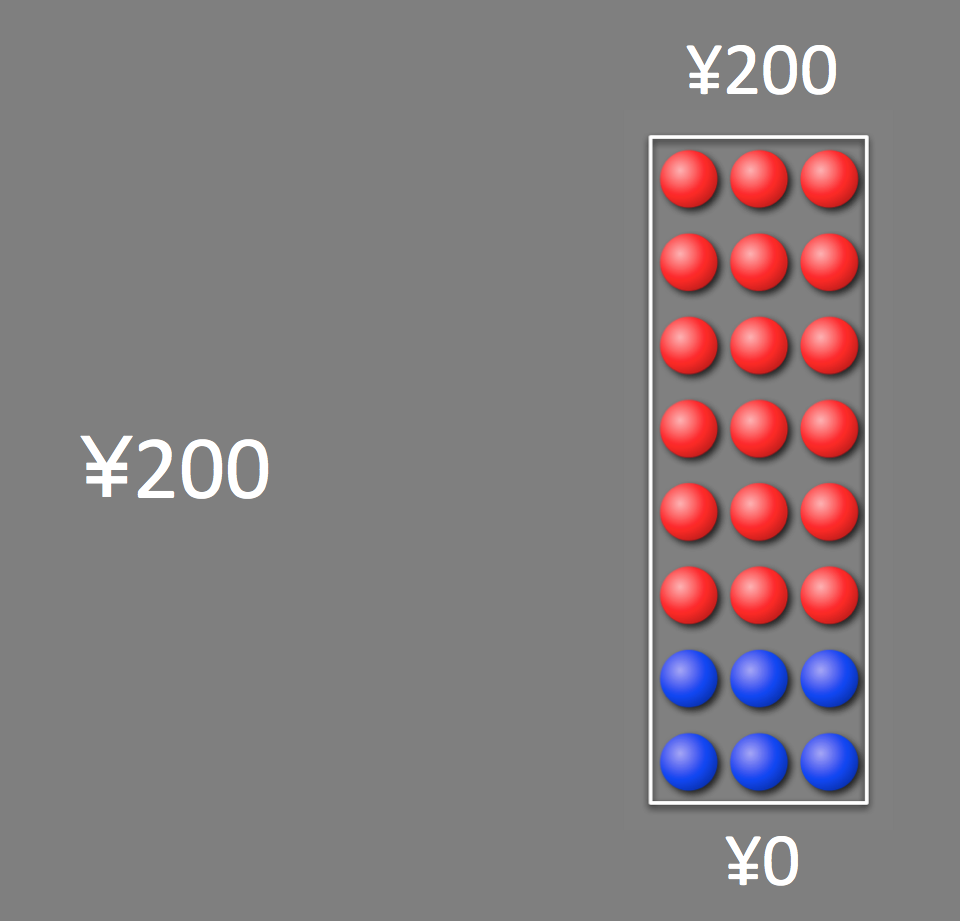


(correct answer [left])

9. Which option is more likely to get you \200 in this trial?


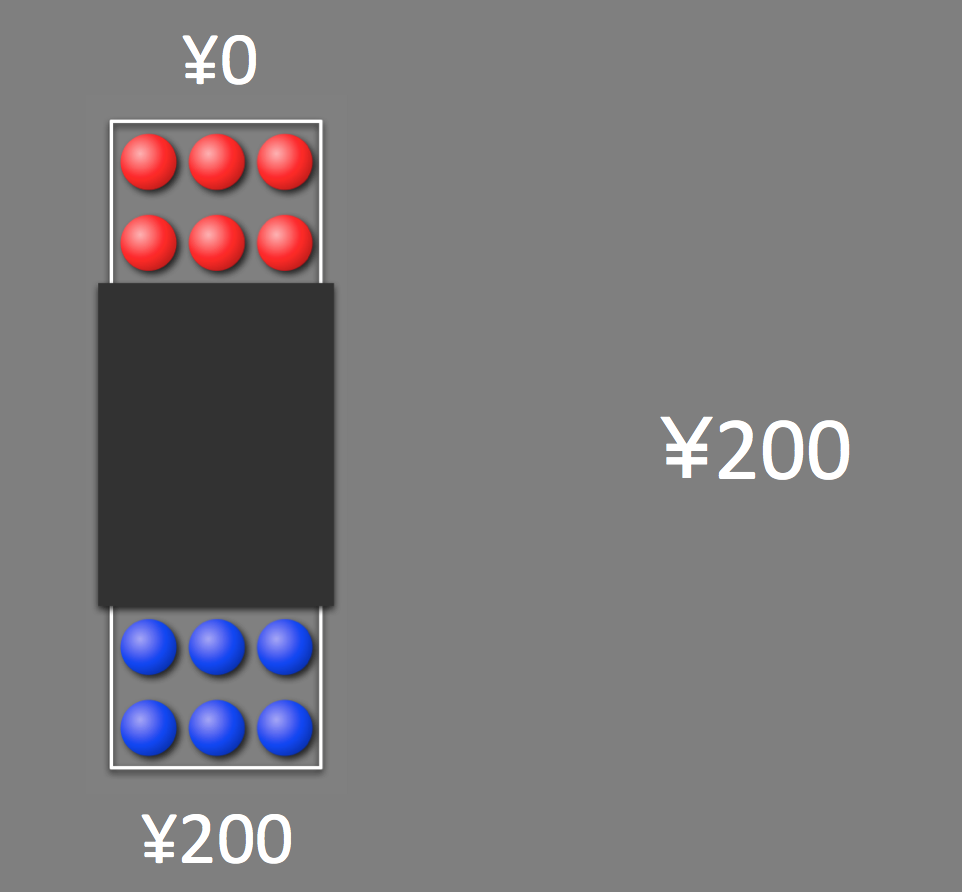


(correct answer [right])

*Quiz for loss trials*

1. How much will you lose if you choose the left side in this trial?


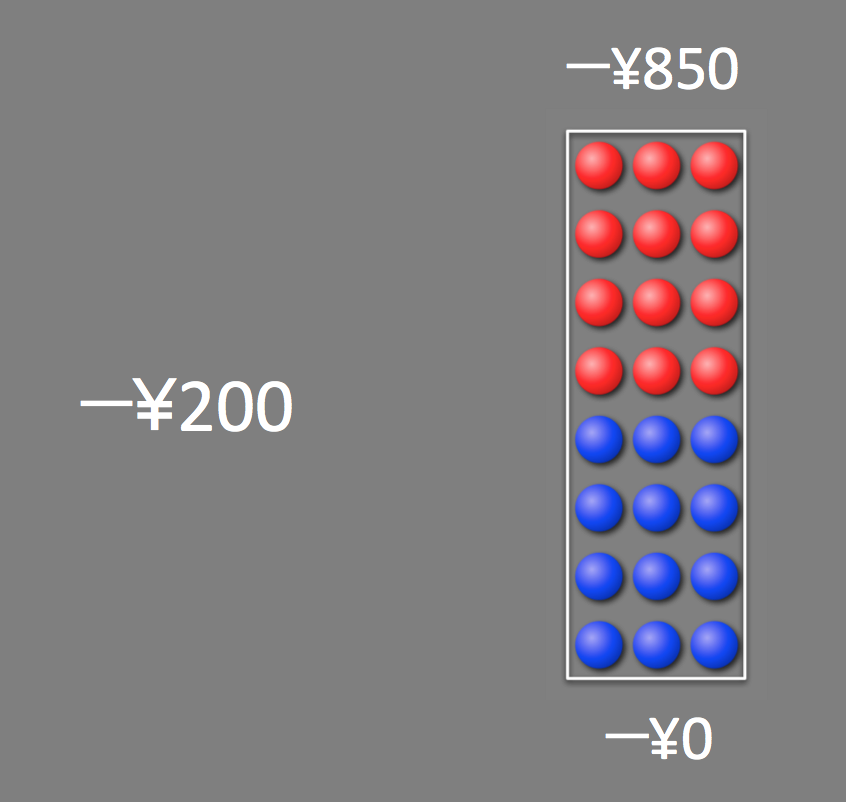


(correct answer [\200])


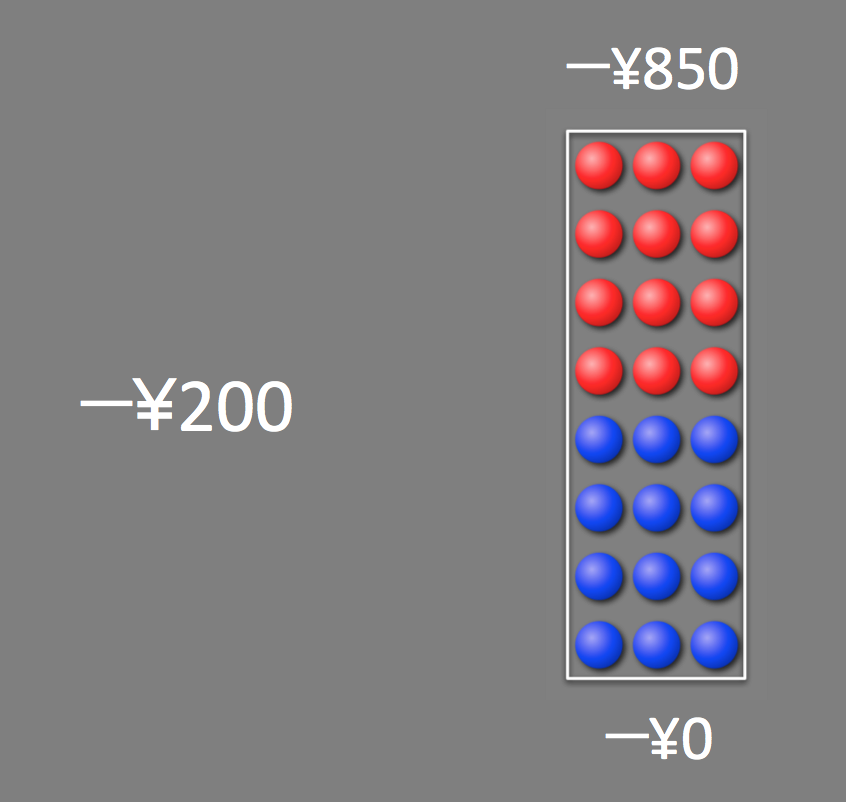
2. How much will you lose if you choose the right side and a red chip is drawn in this trial?

(correct answer [\850])


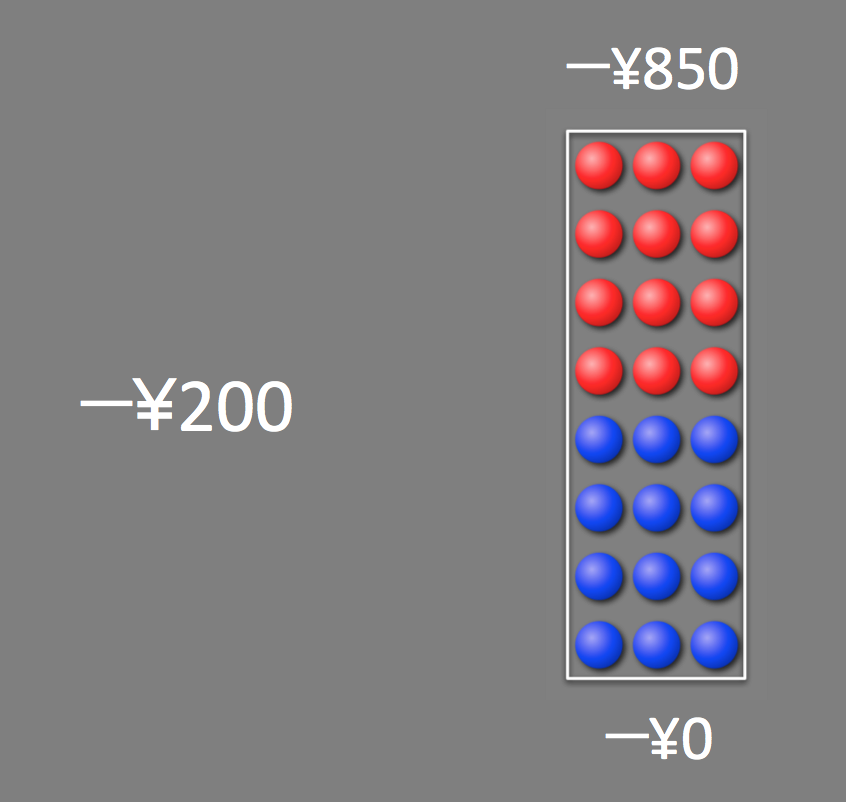
3. How much will you lose if you choose the right side and a blue chip is drawn in this trial?

(correct answer [\0])

4. How much will you lose if you choose the left side and a blue chip is drawn in this trial?


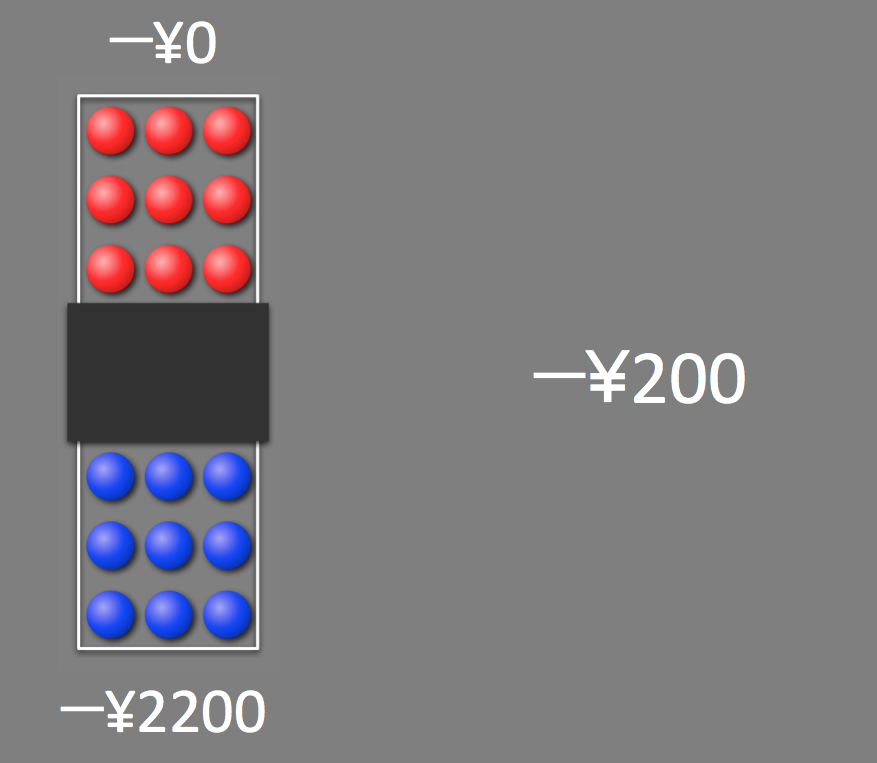


(correct answer [\2200])

5. Which option is more likely to yield a loss in this trial?


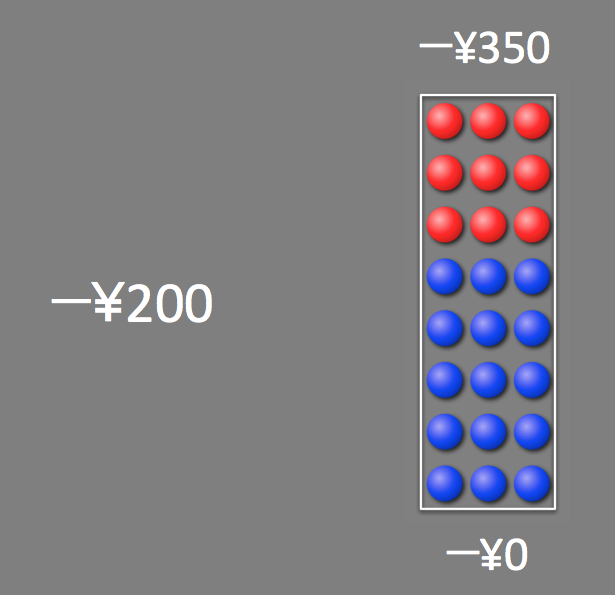


(correct answer [left])

6. Which option is more likely to lose you ¥200 in this trial?


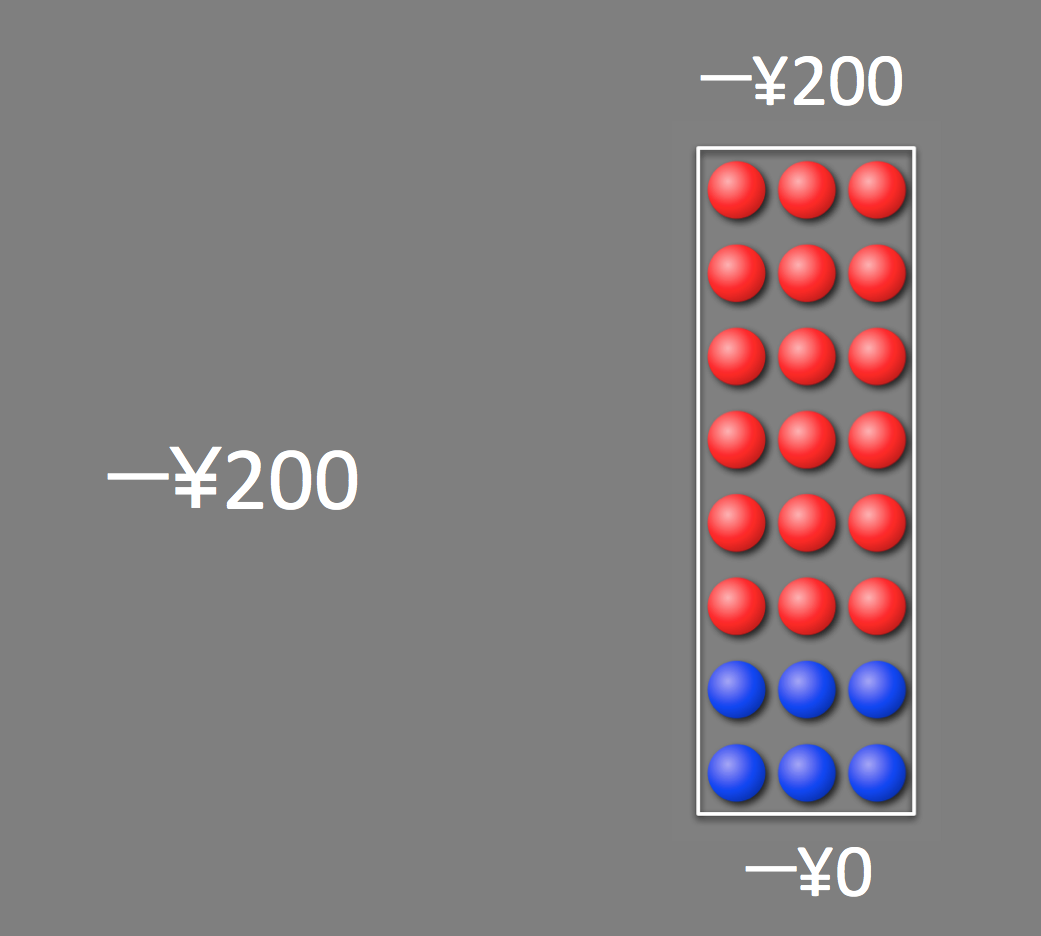


(correct answer [left])

7. Which option is more likely to lose you ¥200 in this trial?


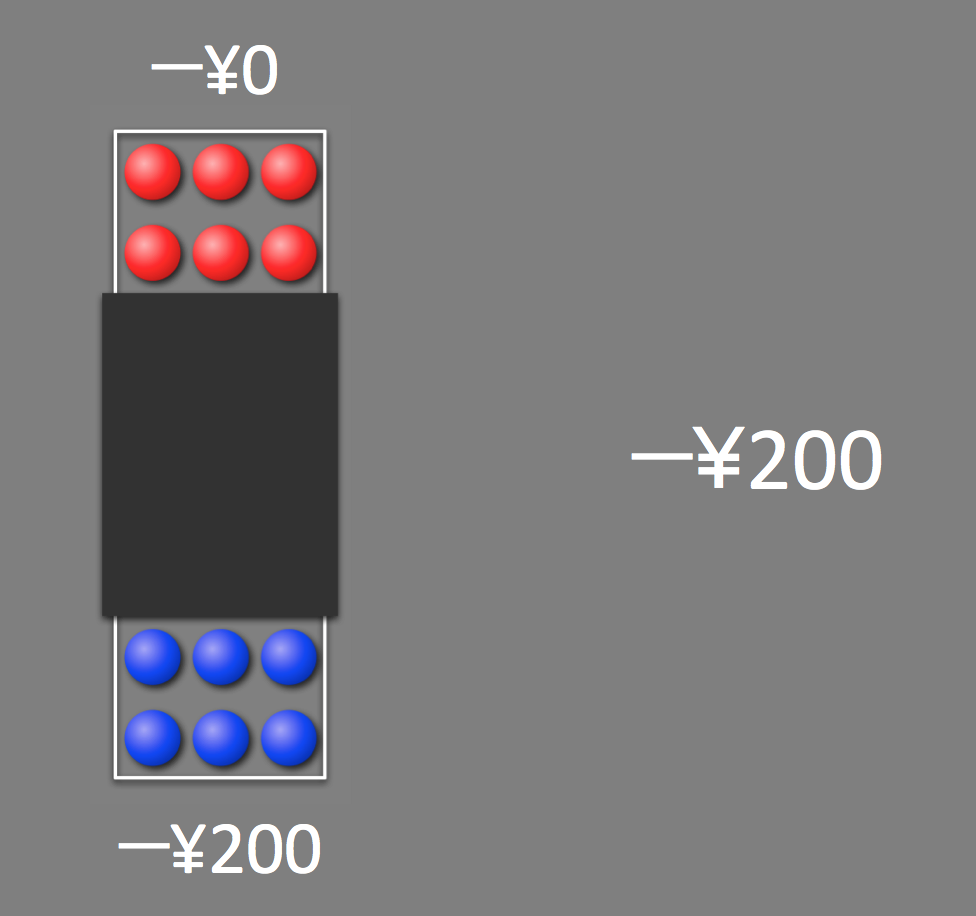


(correct answer [right])

*Decision quality measures*

We measured the decision quality of participants in the gain and loss trial sessions using the two measures mentioned below, which are based on previous research [2, 6-8]. In the first measure, we checked the rate of choosing the inferior option in the gain and loss contexts, respectively.

$$decision quality measure 1 (gain) = \frac{number of uncertain \backslash200 lotteries chosen}{total number of \backslash200 lotteries}$$

$$decision quality measure 1 (loss) = \frac{number of uncertain -\backslash200 lotteries unchosen}{total number of -\backslash200 lotteries}$$

Assuming participants want to maximize their gains and minimize their losses, it is preferable to take a guaranteed gain of \200 over a lottery offering a chance to win \200, and it is preferable to choose the lottery where one can lose ¥200 (but may also lose nothing) over a guaranteed loss of ¥200, regardless of the individual risk and ambiguity attitudes. Therefore, higher scores in this measure indicated poorer decision quality. Based on previous studies [6], we excluded two participants (one control and one ASD) whose measures (gain or loss) were over 0.5 from our analysis because those results suggested that they did not understand the task.

The second measure evaluated each participant’s choice inconsistency by examining their choices on four repetitions of each choice situation. We estimated this by calculating the proportion of choice situations in which participants chose one option on some of the repetitions and another option on the rest of the repetitions. We excluded the choice situations where participants chose between a guaranteed gain (loss) of \200 and \200 (−\200) lottery from this calculation, making it independent of the first measure mentioned above.

$$decision quality measure 2 (gain, loss) = \frac{number of situations where participants behaved inconsistently}{total number of situations}$$

The presence of some inconsistent choices does not necessarily mean choice ineffectiveness. However, an extremely high frequency of such choices (increased scores on this measure) in a given participant would suggest that he or she behaved at random or failed to understand the task. Based on this, we excluded one ASD participant because his measures under gain and loss were both extremely high compared with the average for all the participants (more than 2 SD above the mean)].

**Supplementary Results**

*Distribution of the task measures*

Figure S1 depicts the distribution of each of the task measures (risk attitudes [gain and loss], ambiguity attitudes [gain and loss], and sensitivity to the context change [risk and ambiguity]). Because some of the task measures were not normally distributed (Shapiro–Wilk test, *p* < 0.05), we chose Mann–Whitney tests to compare group differences. Concerning the task measures that were normally distributed, we also compared the group difference using two-sample t-tests, which did not materially change the results. (Figure S1).

*Analyses of the task measures after excluding the potential outliers*

To confirm our conclusion of this study, we also reanalyzed the group comparison of the task measures (risk attitudes [gain and loss], ambiguity attitudes [gain and loss], and sensitivity to the context change [risk and ambiguity]) after excluding the potential outliers of each task measure (>2 SD from the group mean). These analyses did not materially change the results (Figure S2).

*Decision quality in gain and loss trials*

The order of sessions in our task was not counterbalanced (the loss trial session followed the gain trial session for all participants). To objectively/precisely address this issue, we performed the following additional analyses using measures related to comprehension/attention (decision quality).

1. To compare participants’ decision quality between gain and loss trials in both groups, we performed the following comparisons. Wilcoxon tests were used.

(Controls)

1. Number of missing trials (gain trials) vs. number of missing trials (loss trials)

2. Decision quality measure 1 (gain trials) vs. decision quality measure 1 (loss trials)

3. Decision quality measure 2 (gain trials) vs. decision quality measure 2 (loss trials)

(ASD)

4. Number of missing trials (gain trials) vs. number of missing trials (loss trials)

5. Decision quality measure 1 (gain trials) vs. decision quality measure 1 (loss trials)

6. Decision quality measure 2 (gain trials) vs. decision quality measure 2 (loss trials)

1. To compare participants’ decision quality between the groups during gain and loss trials, we performed the following comparisons (no significant differences in decision quality measures 1 and 2 were also shown in Additional file 2: Table S5). Mann-Whitney tests were used.

(Gain trials)

7. Number of missing trials (controls) vs. number of missing trials (ASD)

8. Decision quality measure 1 (controls) vs. decision quality measure 1 (ASD)

9. Decision quality measure 2 (controls) vs. decision quality measure 2 (ASD)

(Loss trials)

10. Number of missing trials (controls) vs. number of missing trials (ASD)

11. Decision quality measure 1 (controls) vs. decision quality measure 1 (ASD)

12. Decision quality measure 2 (controls) vs. decision quality measure 2 (ASD)

1. To assess the effect of time on the participants’ decision quality, we divided gain trials into the former and latter halves. Similarly, we also divided loss trials into the former and latter halves. Subsequently, we performed the following comparisons. Decision quality measure 2 calculations required all trials; therefore, this measure was not included in the analysis. Wilcoxon tests were used.

(Controls, gain trials)

13. Number of missing trials (former half) vs. number of missing trials (latter half)

14. Decision quality measure 1 (former half) vs. decision quality measure 1 (latter half)

(Controls, loss trials)

15. Number of missing trials (former half) vs. number of missing trials (latter half)

16. Decision quality measure 1 (former half) vs. decision quality measure 1 (latter half)

(ASD, gain trials)

17. Number of missing trials (former half) vs. number of missing trials (latter half)

18. Decision quality measure 1 (former half) vs. decision quality measure 1 (latter half)

(ASD, loss trials)

19. Number of missing trials (former half) vs. number of missing trials (latter half)

20. Decision quality measure 1 (former half) vs. decision quality measure 1 (latter half)

We found no significant difference in any of the above comparisons (1–20) (for all, *p* > 0.05, two tailed). Regarding the measures that were normally distributed, we also reanalyzed the data using two-sample [paired (a, c) or unpaired (b)] t-tests. These analyses did not materially change the results (for all, *p* > 0.05, two tailed).

**Supplementary References**

1. Levy I, Snell J, Nelson AJ, Rustichini A, Glimcher PW. Neural representation of subjective value under risk and ambiguity. J Neurophysiol. 2010;103:1036-47.

2 Tymula A, Belmaker LAR, Ruderman L, Glimcher PW, Levy I. Like cognitive function, decision making across the life span shows profound age-related changes. Proc Natl Acad Sci USA. 2013;110:17143-8.

3. Bernoulli, D. Exposition of a new theory on the measurement of risk. Econometrica. 1954;22:23-36.

4. Camerer C, Weber M. Recent developments in modeling preferences: Uncertainty and ambiguity. J Risk Uncertainty. 1992;5:325-70.

5. Ellsberg, D. Risk, ambiguity, and the Savage axioms. Q J Econ. 1961;75:643-69.

6. Ruderman L, Ehrlich DB, Roy A, Pietrzak RH, Harpaz‐Rotem I, Levy I. Posttraumatic stress symptoms and aversion to ambiguous losses in combat veterans. Depress Anxiety. 2016;33:606-13.

7. Tymula A, Belmaker LAR, Roy AK, Ruderman L, Manson K, Glimcher PW, et al. Adolescents’ risk-taking behavior is driven by tolerance to ambiguity. Proc Natl Acad Sci USA. 2012, 109;17135-40.

8. Pushkarskaya H, Tolin D, Ruderman L, Kirshenbaum A, Kelly JM, Pittenger C, et al. Decision-making under uncertainty in obsessive–compulsive disorder. J Psychiatr Res. 2015;69:166-73.
