# Supplementary material for: Attitudes toward risk and ambiguity in patients with autism spectrum disorder
Source: Mol Autism. 2017 Aug 16;8:45. doi: 10.1186/s13229-017-0162-8 (PMC5559781; doi:10.1186/s13229-017-0162-8)
Supplement: Supplementary file 2 — Tables showing the details of the task measures. Table S1. All lottery situations in the gain trials. Table S2. All lottery situations in the loss trials. Table S3. Probability of choosing a lottery for a risk-neutral decision maker in the gain contexts. Table S4. Probability of choosing a lottery for a risk-neutral decision maker in the loss contexts. Table S5. Measures of decision quality. Table S6. Reaction time (ms) in risk and ambiguity conditions. Table S7. Results of additional analyses between ASD participants without psychotropic medication and controls. Table S8. Correlation coefficients between risk and ambiguity attitudes and the three factors of the Autism-Spectrum Quotient (AQ) among ASD participants. (DOCX 84 kb) [file 13229_2017_162_MOESM2_ESM.docx]

**Additional file 2**

**Table S1. All lottery situations in the gain trials**

| ***Risk*** | |
| --- | --- |
| Winning amount | Winning probability |
| \200 | 0.125 |
| \200 | 0.25 |
| \200 | 0.375 |
| \200 | 0.5 |
| \200 | 0.75 |
| \350 | 0.125 |
| \350 | 0.25 |
| \350 | 0.375 |
| \350 | 0.5 |
| \350 | 0.75 |
| \850 | 0.125 |
| \850 | 0.25 |
| \850 | 0.375 |
| \850 | 0.5 |
| \850 | 0.75 |
| \2200 | 0.125 |
| \2200 | 0.25 |
| \2200 | 0.375 |
| \2200 | 0.5 |
| \2200 | 0.75 |
| \5200 | 0.125 |
| \5200 | 0.25 |
| \5200 | 0.375 |
| \5200 | 0.5 |
| \5200 | 0.75 |

| ***Ambiguity*** | |
| --- | --- |
| Winning amount | Ambiguity level |
| \200 | 0.25 |
| \200 | 0.5 |
| \200 | 0.75 |
| \350 | 0.25 |
| \350 | 0.5 |
| \350 | 0.75 |
| \850 | 0.25 |
| \850 | 0.5 |
| \850 | 0.75 |
| \2200 | 0.25 |
| \2200 | 0.5 |
| \2200 | 0.75 |
| \5200 | 0.25 |
| \5200 | 0.5 |
| \5200 | 0.75 |

**Table S2. All lottery situations in the loss trials**

| ***Risk*** | |
| --- | --- |
| Losing amount | Losing probability |
| −\200 | 0.125 |
| −\200 | 0.25 |
| −\200 | 0.375 |
| −\200 | 0.5 |
| −\200 | 0.75 |
| −\250 | 0.125 |
| −\250 | 0.25 |
| −\250 | 0.375 |
| −\250 | 0.5 |
| −\250 | 0.75 |
| −\350 | 0.125 |
| −\350 | 0.25 |
| −\350 | 0.375 |
| −\350 | 0.5 |
| −\350 | 0.75 |
| −\850 | 0.125 |
| −\850 | 0.25 |
| −\850 | 0.375 |
| −\850 | 0.5 |
| −\850 | 0.75 |
| −\2200 | 0.125 |
| −\2200 | 0.25 |
| −\2200 | 0.375 |
| −\2200 | 0.5 |
| −\2200 | 0.75 |

| ***Ambiguity*** | |
| --- | --- |
| Losing amount | Ambiguity level |
| −\200 | 0.25 |
| −\200 | 0.5 |
| −\200 | 0.75 |
| −\250 | 0.25 |
| −\250 | 0.5 |
| −\250 | 0.75 |
| −\350 | 0.25 |
| −\350 | 0.5 |
| −\350 | 0.75 |
| −\850 | 0.25 |
| −\850 | 0.5 |
| −\850 | 0.75 |
| −\2200 | 0.25 |
| −\2200 | 0.5 |
| −\2200 | 0.75 |

**Table S3. Probability of choosing a lottery for a risk-neutral decision maker in the gain contexts**

| Probability | Amount | | | | |
| --- | --- | --- | --- | --- | --- |
|  | \200 | \350 | \850 | \2200 | \5200 |
| 0.125 | 0 | 0 | 0 | 1 | 1 |
| 0.25 | 0 | 0 | 1 | 1 | 1 |
| 0.375 | 0 | 0 | 1 | 1 | 1 |
| 0.5 | 0 | 0 | 1 | 1 | 1 |
| 0.75 | 0 | 1 | 1 | 1 | 1 |

Across all choices, a risk-neutral decision maker would choose a lottery 60% of the time in gain contexts.

**Table S4. Probability of choosing a lottery for a risk-neutral decision maker in the loss contexts**

| Probability | Amount | | | | |
| --- | --- | --- | --- | --- | --- |
|  | −\200 | −\250 | −\350 | −\850 | −\2200 |
| 0.125 | 1 | 1 | 1 | 1 | 0 |
| 0.25 | 1 | 1 | 1 | 0 | 0 |
| 0.375 | 1 | 1 | 1 | 0 | 0 |
| 0.5 | 1 | 1 | 1 | 0 | 0 |
| 0.75 | 1 | 1 | 0 | 0 | 0 |

Across all choices, a risk-neutral decision maker would choose a lottery 60% of the time in loss contexts.

**Table S5. Measures of decision quality**

|  | Control Group |  | ASD Group |  | Statistics |
| --- | --- | --- | --- | --- | --- |
|  | (*N* = 26) |  | (*N* = 24) |  | *p* |
| Decision quality measure 1 (gain) | 0.01 (0.03) |  | 0.01 (0.02) |  | 0.94 |
| Decision quality measure 2 (gain) | 0.22 (0.11) |  | 0.18 (0.12) |  | 0.32 |
| Decision quality measure 1 (loss) | 0.03 (0.06) |  | 0.03 (0.06) |  | 0.54 |
| Decision quality measure 2 (loss) | 0.24 (0.12) |  | 0.23 (0.15) |  | 0.59 |

Standard deviations are given in parentheses

**Table S6. Reaction time (ms) in risk and ambiguity conditions**

|  | Control Group |  | ASD Group |  | Statistics |
| --- | --- | --- | --- | --- | --- |
|  | (*N* = 26) |  | (*N* = 24) |  | *p* |
| Risk (gain) | 1163.6 (299.8) |  | 1144.5 (244.6) |  | 0.97 |
| Risk (loss) | 1272.2 (342.5) |  | 1339.8 (298.3) |  | 0.28 |
| Ambiguity (gain) | 1286.3 (342.1) |  | 1220.0 (325.3) |  | 0.50 |
| Ambiguity (loss) | 1346.7 (508.5) |  | 1407.8 (397.1) |  | 0.35 |

Standard deviations are given in parentheses

**Table S7. Results of additional analyses between ASD participants without psychotropic medication and controls**

|  | Control |  | ASD |  | Statistics | | | | | | |
| --- | --- | --- | --- | --- | --- | --- | --- | --- | --- | --- | --- |
|  | (*N* = 26) |  | (*N* = 12) |  | *U* |  | *Z* |  | *p* |  | *r* (effect size) |
| Risk attitudes (gain) | 0.25 (0.17) |  | 0.34 (0.18) |  | 105.50 |  | −1.59 |  | 0.11 |  | −0.26 |
| Risk attitudes (loss) | −0.06 (0.12) |  | 0.04 (0.12) |  | 77.00 |  | −2.48 |  | 0.01 |  | −0.40 |
| Sensitivity to the context change (risk) | 0.32 (0.19) |  | 0.29 (0.19) |  | 146.00 |  | −0.31 |  | 0.75 |  | −0.05 |
| Ambiguity attitudes (gain) | 0.18 (0.18) |  | 0.06 (0.12) |  | 92.50 |  | −2.00 |  | 0.045 |  | −0.32 |
| Ambiguity attitudes (loss) | 0.01 (0.11) |  | 0.05 (0.09) |  | 130.50 |  | −0.80 |  | 0.42 |  | −0.13 |
| Sensitivity to the context change (ambiguity) | 0.16 (0.18) |  | 0.02 (0.09) |  | 75.00 |  | −2.55 |  | 0.01 |  | −0.41 |

Standard deviations are given in parentheses

**Table S8. Correlation coefficients between risk and ambiguity attitudes and the three factors of the Autism-Spectrum Quotient (AQ) among ASD participants**

|  | Social skills | Details/patterns | Communication  /mindreading |
| --- | --- | --- | --- |
| Risk attitudes (gain) | −0.29 (0.17) | −0.12 (0.57) | −0.43 (0.03)* |
| Risk attitudes (loss) | 0.15 (0.49) | −0.37 (0.07) | −0.33 (0.11) |
| Sensitivity to the context change (risk) | −0.44 (0.03)* | 0.26 (0.21) | −0.12 (0.58) |
| Ambiguity attitudes (gain) | 0.04 (0.85) | 0.09 (0.67) | 0.30 (0.15) |
| Ambiguity attitudes (loss) | 0.37 (0.08) | −0.16 (0.46) | 0.06 (0.78) |
| Sensitivity to the context change (ambiguity) | −0.50 (0.01)* | 0.40 (0.054) | 0.25 (0.23) |

The *p* values are given in parentheses; **p* < 0.05; *N* (ASD) = 24
