# Supplementary figures and images for: Attitudes toward risk and ambiguity in patients with autism spectrum disorder
Source: Mol Autism. 2017 Aug 16;8:45. doi: 10.1186/s13229-017-0162-8 (PMC5559781; doi:10.1186/s13229-017-0162-8)

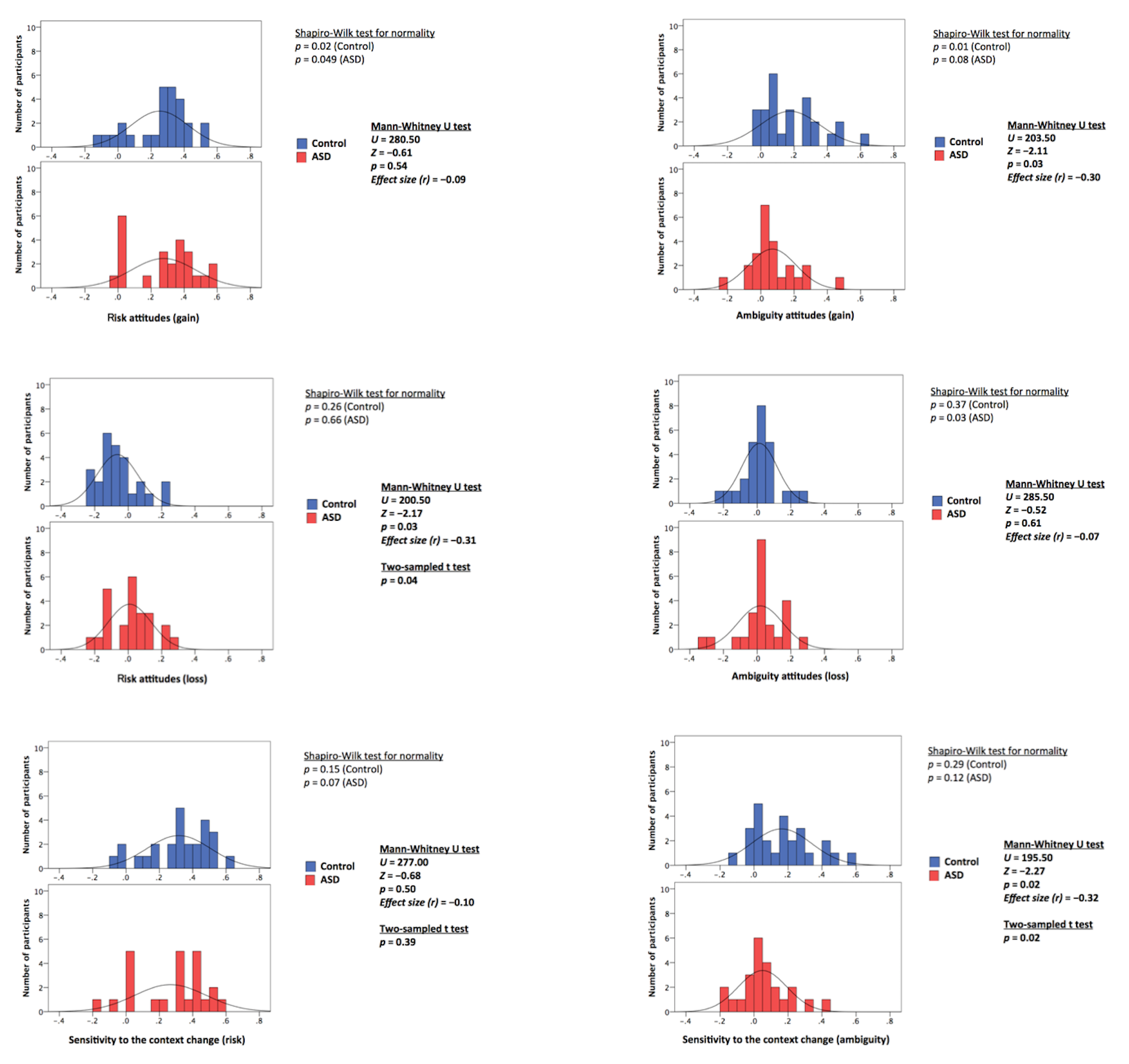

Supplement: Supplementary file 3 — Distribution of the task measures (Figure S1.) Figure S1. depicts the distribution of each of the task measures (risk attitudes [gain and loss], ambiguity attitudes [gain and loss], and sensitivity to the context change [risk and ambiguity]). Because some of the task measures were not normally distributed (Shapiro–Wilk test, p < 0.05), we chose Mann–Whitney tests to compare group differences. Concerning the task measures that were normally distributed, we also compared the group difference using two-sample t-tests, which did not materially change the results. (TIFF 1149 kb) [file 13229_2017_162_MOESM3_ESM.tiff]

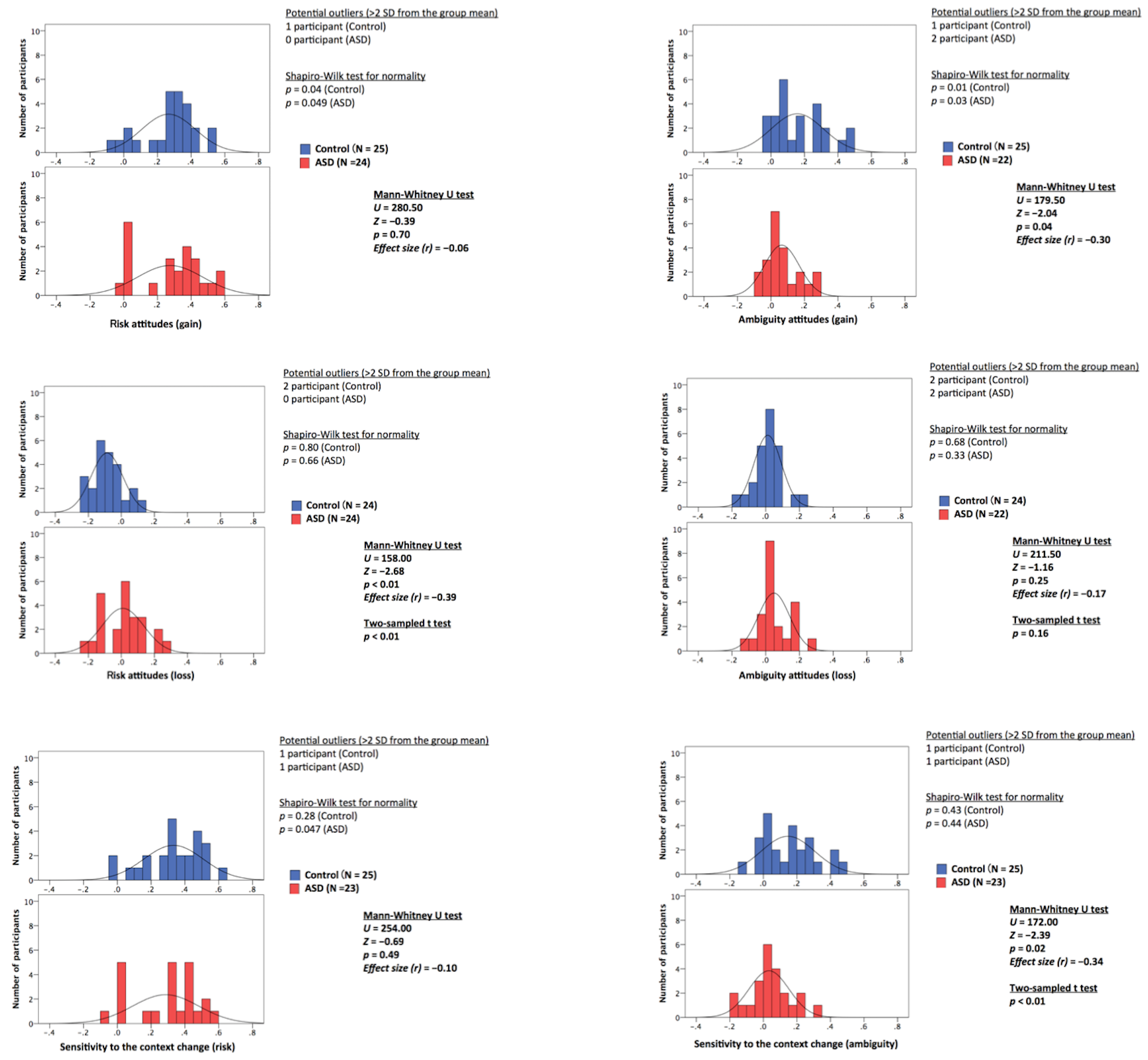

Supplement: Supplementary file 4 — Analyses of the task measures after excluding the potential outliers (Figure S2.) To confirm our conclusion of this study, we also reanalyzed the group comparison of the task measures (risk attitudes [gain and loss], ambiguity attitudes [gain and loss], and sensitivity to the context change [risk and ambiguity]) after excluding the potential outliers of each task measure (> 2 SD from the group mean). These analyses did not materially change the results. (TIFF 1368 kb) [file 13229_2017_162_MOESM4_ESM.tiff]
